# Long-read sequencing for non-small-cell lung cancer genomes

**DOI:** 10.1101/gr.261941.120

**Published:** 2020-09

**Authors:** Yoshitaka Sakamoto, Liu Xu, Masahide Seki, Toshiyuki T. Yokoyama, Masahiro Kasahara, Yukie Kashima, Akihiro Ohashi, Yoko Shimada, Noriko Motoi, Katsuya Tsuchihara, Susumu S. Kobayashi, Takashi Kohno, Yuichi Shiraishi, Ayako Suzuki, Yutaka Suzuki

**Affiliations:** 1 Department of Computational Biology and Medical Sciences, Graduate School of Frontier Sciences, The University of Tokyo, Chiba 277-8562, Japan;; 2 Division of Translational Informatics, Exploratory Oncology Research and Clinical Trial Center, National Cancer Center, Chiba 277-8577, Japan;; 3 Division of Translational Genomics, Exploratory Oncology Research and Clinical Trial Center, National Cancer Center, Chiba 277-8577, Japan;; 4 Division of Genome Biology, National Cancer Center Research Institute, Tokyo 104-0045, Japan;; 5 Department of Pathology, National Cancer Center Hospital, Tokyo 104-0045, Japan;; 6 Division of Cellular Signaling, National Cancer Center Research Institute, Tokyo 104-0045, Japan

## Abstract

Here, we report the application of a long-read sequencer, PromethION, for analyzing human cancer genomes. We first conducted whole-genome sequencing on lung cancer cell lines. We found that it is possible to genotype known cancerous mutations, such as point mutations. We also found that long-read sequencing is particularly useful for precisely identifying and characterizing structural aberrations, such as large deletions, gene fusions, and other chromosomal rearrangements. In addition, we identified several medium-sized structural aberrations consisting of complex combinations of local duplications, inversions, and microdeletions. These complex mutations occurred even in key cancer-related genes, such as *STK11*, *NF1*, *SMARCA4*, and *PTEN*. The biological relevance of those mutations was further revealed by epigenome, transcriptome, and protein analyses of the affected signaling pathways. Such structural aberrations were also found in clinical lung adenocarcinoma specimens. Those structural aberrations were unlikely to be reliably detected by conventional short-read sequencing. Therefore, long-read sequencing may contribute to understanding the molecular etiology of patients for whom causative cancerous mutations remain unknown and therapeutic strategies are elusive.

Recent cancer sequencing projects, such as the International Cancer Genome Consortium (ICGC) and The Cancer Genome Atlas (TCGA), have revealed causative mutations in various types of cancer (The International Cancer Genome Consortium 2010; The Cancer Genome Atlas Research Network et al. 2013). Among them, lung adenocarcinomas are one of the most well-studied cancers regarding such “cancer driver” mutations (Imielinski et al. 2012; Seo et al. 2012; Suzuki et al. 2013a; The Cancer Genome Atlas Research Network 2014). More than half of the patients with lung cancer have characteristic point mutations in the *EGFR* and *KRAS* genes or gene fusions in the *ALK*, *RET*, and *ROS1* genes. These mutations are used as “biomarkers,” which provide fundamental information about the most appropriate therapeutic strategy. Patients are separated based on their genomic mutation statuses and are matched accordingly to the most appropriate treatment (Kohno et al. 2013; Yoh et al. 2016; Mainardi et al. 2018; Ruess et al. 2018; Wong et al. 2018). Despite the general success of this approach, ∼20%–30% of patients with lung adenocarcinoma remain undiagnosed with respect to their cancerous mutations (Kohno et al. 2013).

Current information on cancer-related mutations has mostly been obtained by short-read sequencing. Short-read sequencing data, generally consisting of tens of millions of reads of up to 200–300 bases in length (Treangen and Salzberg 2012), are the most powerful for detecting point mutations such as single-nucleotide variants (SNVs) and short indels (Pleasance et al. 2010; Suzuki et al. 2013a). Significant efforts have been made to enable the identification of fusion genes by using short-read sequences (Liu et al. 2015). However, it is still difficult to detect more complex or larger-scale structural aberrations, such as chromosome aneuploidy, copy-number aberrations, and rearrangements, based solely on short-read sequencing data. There are inherent drawbacks even in the latest bioinformatics pipelines for this purpose, which hampers their practical use without careful validation (Guan and Sung 2016).

Recently developed long-read sequencing technologies are changing this situation. Several pioneering studies have reported the precise analysis of complicated genomic regions, and large-scale aberration detection is enabled by long-read sequencing. For example, a single-molecule real-time (SMRT) sequencer, Pacific Biosciences (PacBio) RS, has been used to analyze *BCR-ABL1* rearranged transcripts and their TKI-resistant mutations in chronic myeloid leukemia (Cavelier et al. 2015; Ardui et al. 2018). In chromophobe renal cell carcinoma, structural alterations in the *TERT* promoter region have been identified and characterized by PacBio sequencing (Davis et al. 2014). In 2018, a nanopore-type sequencer, MinION, was first used to characterize the pathogenic sequence expansion of intronic repeats in benign adult familial myoclonic epilepsy (Ishiura et al. 2018). Particularly for cancer applications, we and others have shown that cancer-associated structural variants (SVs) could be detected by long-read sequencing approaches (Norris et al. 2016; Suzuki et al. 2017; Nattestad et al. 2018). Indeed, a pioneering study by Nattestad et al. (2018) reported the first whole-genome sequencing of a breast cancer cell line, SK-BR-3, using a PacBio sequencer. They also developed a series of computational programs, including Sniffles to detect SVs from long-read sequencing data (Sedlazeck et al. 2018). Additionally, transcriptome sequencing by MinION has been shown to provide a powerful analytical platform, where the complete splicing pattern of a given mRNA can be thoroughly represented by a single read (Oikonomopoulos et al. 2016). Further improvements in MinION sequencing have been achieved by the parallelization of nanopores in a given flow cell, a platform named PromethION, which can now produce >100 Gb of data per flow cell.

In this study, we attempted long-read sequencing of whole human cancer genomes by using PromethION. We first show that PromethION sequencing can identify point mutations, as well as large structural aberrations and fusion genes relatively easily. Moreover, we identified that mutations containing complex combinations of small-sized and medium-sized structural aberrations are highly common, constituting a previously undefined unique class of mutations. We initially used lung cancer cell lines for which we had previously collected detailed information on multiomics features, such as whole-genome sequencing, RNA-seq, and ChIP-seq of Illumina reads (Suzuki et al. 2014). Then, we used clinical samples to show that those complicated SVs are not restricted to cell lines.

## Results

### Long-read sequencing of cancer cell lines

**Figure 1.**
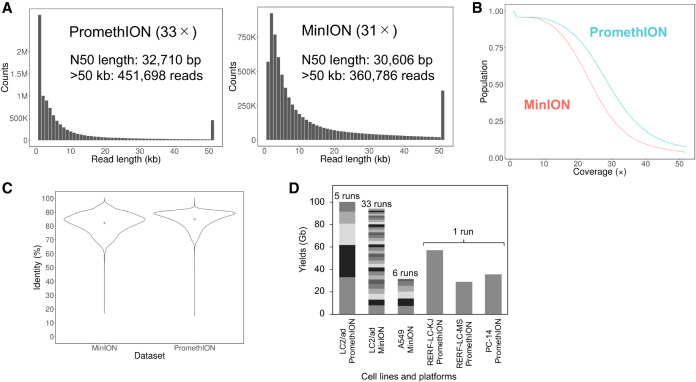
Long-read sequencing of cancer genomes. (*A*, *left*) Raw read length distribution of LC/2ad PromethION sequencing. (*Right*) Raw read length distribution of LC2/ad MinION sequencing. Both PromethION and MinION data sets have many long reads (e.g., >50 kb, 452 kilo reads, and 361 kilo reads, respectively). (*B*) Cumulative depth curve of LC2/ad. Blue indicates PromethION; red, MinION. More than 50% of the human genome was covered by a sequencing depth of >20× in both data sets. (*C*) Violin plots of sequence identity of LC2/ad MinION sequencing (*left*) and PromethION sequencing (*right*). The points in the violin plots indicate the mean identities of the MinION and PromethION data (82.1% and 84.8%, respectively) (Table 1). The identities were concentrated at >80% in both data sets. (*D*) Sequencing yields for each data set of PromethION and MinION. Yields from individual flow cells are represented by different colors.

We conducted a long-read and whole-genome sequencing analysis using the nanopore-type sequencers MinION and its latest high-throughput derivative PromethION. To evaluate the performance of PromethION, we first performed whole-genome sequencing of the genome of LC2/ad, a lung cancer cell line derived from a Japanese patient with lung adenocarcinoma (Matsubara et al. 2012; Suzuki et al. 2013b). We generated a total of more than 10 million reads (>100 Gb) with overall coverage of 33× using five flow cells (Fig. 1; for general sequencing statistics of one PromethION flow cell, see Supplemental Fig. S1). The maximum length and N50 length of reads were 987,834 and 32,710 bp, respectively. By using minimap2 (Li 2018), we mapped 69% of the reads to the reference genome UCSC hg38. The average length of the mapped reads was 13,620 bp, and the average identity was 85% (Table 1). As a reference of LC2/ad data from long-read sequencing, we also collected the whole-genome sequencing data from a total of 33 MinION runs (R9.5 flow cells) to cover the whole human genome at an overall sequencing depth of 31× by a total of more than 7 million reads (>90 Gb). The maximum length and N50 length of the reads were >2 Mb and exactly 30,606 bp, respectively. In total, 68% of the reads were mapped to the human reference genome by using minimap2. The calculated overall sequence identity was 82% on average. The average length of the mapped reads was 16,452 bp, which was significantly longer than previous long-read whole human cancer genome sequencing analyses (Table 1; Seo et al. 2016; Shi et al. 2016; Mizuguchi et al. 2019). Because sample preparation need not be performed for each run, the required total amount of starting DNA used for PromethION could be reduce to less than one-tenth compared with that for MinION.

**Table 1.**
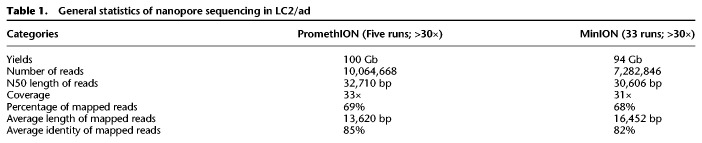
General statistics of nanopore sequencing in LC2/ad

To examine whether PromethION sequencing was compatible with MinION sequencing, we compared the features of the two obtained data sets. The overall distributions of read lengths were similar (Fig. 1A). Both data sets included a substantial fraction of long reads >50 kb (MinION: 360,786 reads, PromethION: 451,698 reads). Detailed analysis of the mapping results showed that >50% of the human genome was covered at >20× sequencing depth in both of the data sets (Fig. 1B). Regarding the sequence accuracy, both data sets showed overall fidelity of >80% (Fig. 1C), which is similar to that of a previous study (Jain et al. 2018). We concluded that PromethION should be an effective analytical method for whole cancer genome sequencing.

### Application of long-read sequencing for analyzing the lung cancer genomes

After initial evaluation of the data obtained from MinION and PromethION from LC2/ad cells, we scaled the MinION or PromethION sequencing for an additional four lung cancer cell lines (MinION data from A549; PromethION data from RERF-LC-KJ, RERF-LC-MS, and PC-14 cells) (for detailed cellular profiles, see Supplemental Table S1). The data production proceeded similarly to the case of LC2/ad cells; for example, in the sequencing of RERF-LC-KJ, about six million reads were generated (>57 Gb, at 19×), with the maximum and N50 lengths of the reads being 922,768 and 23,442 bp, respectively (Fig. 1D). Other detailed statistics are shown in Figure 1D, Supplemental Figure S2, and Supplemental Table S2.

We particularly focused on the SNVs in the most well-known cancer driver mutations by using Nanopolish (Loman et al. 2015). To further confirm the results, the detected SNVs were also viewed by using Integrative Genomics Viewer (IGV) (Robinson et al. 2011; Thorvaldsdóttir et al. 2013). In A549, 11 reads illustrated the cancerous mutation *KRAS* G12S as the point mutation (Supplemental Fig. S3A, left). In PC-14, eight reads represented the driver *NRAS* Q61K point mutation (Supplemental Fig. S3A, right). Conversely, we also confirmed the absence of any driver mutations in the RERF-LC-KJ and RERF-LC-MS cell lines at well-known driver genes. All these results are consistent with those of previous reports (Suzuki et al. 2014). Additional detailed evaluations of SNV detection are shown in Supplemental Figure S3, B through D.

### Detection of cancerous SVs using the PromethION long reads

**Figure 2.**
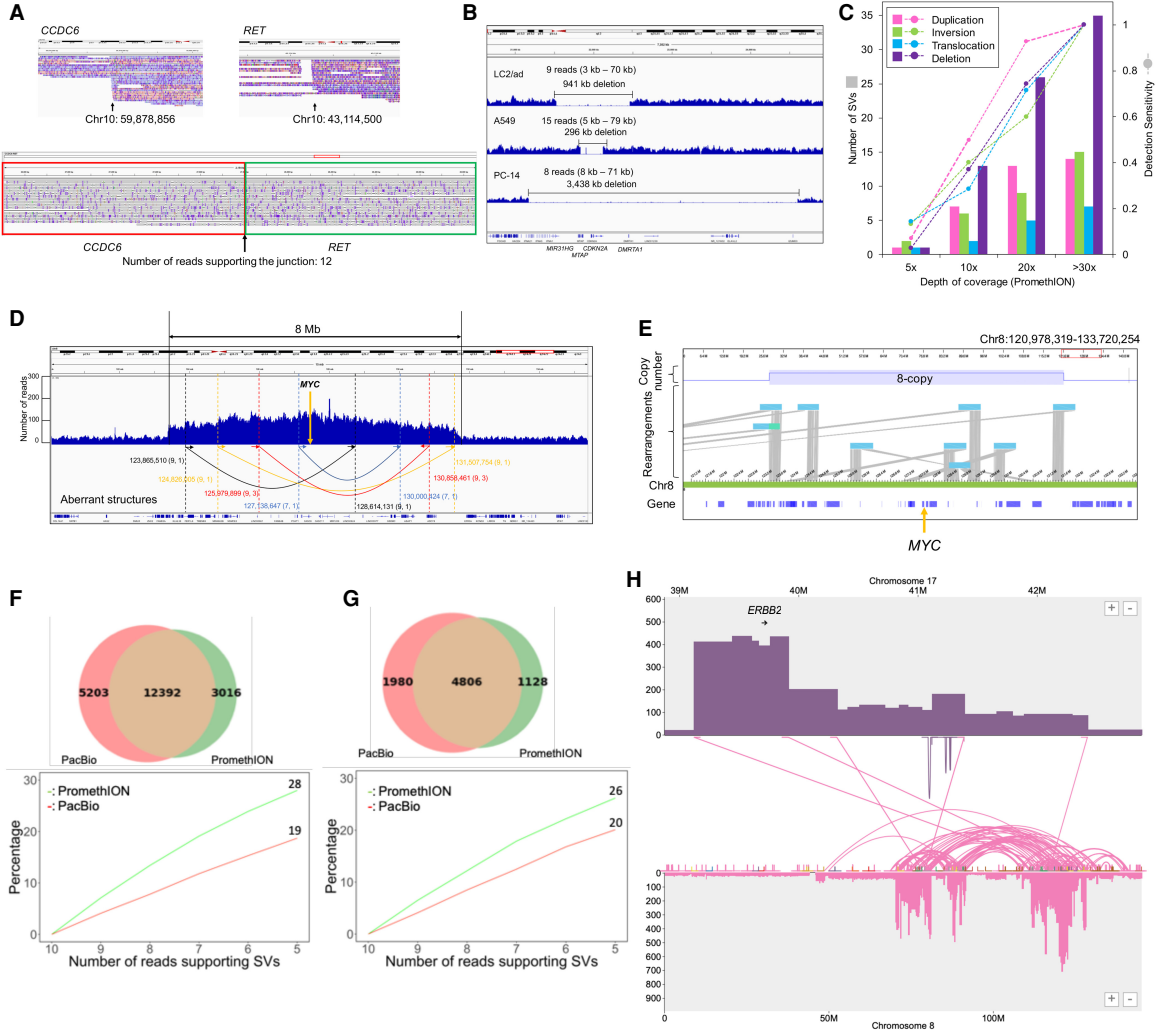
Structural variant analysis of cancer genomes. (*A*) IGV image of the *CCDC6-RET* fusion gene of LC2/ad MinION sequencing. The *CCDC6-RET* fusion gene is a driver mutation of LC2/ad cells. The number of reads supporting the junction is 12. (*B*) IGV image of a large deletion including *CDKN2A* of LC2/ad, A549, and PC-14. The deletion of LC2/ad spanned ∼941 kb. The deletion of A549 spanned ∼296 kb. The deletion of PC-14 spanned ∼3438 kb. The parentheses indicate the range of length of reads supporting the deletions. (*C*) The number of SVs and sensitivities of SV detection in each depth of coverage of PromethION reads. We randomly selected reads to collectively sum up to the indicated sequencing coverage. (*D*) Depth plotting around the *MYC* gene of LC2/ad. Arrows indicate junction candidates of the amplification supported by MinION and Illumina reads. Parentheses indicate the number of supporting MinION reads (*left*) and Illumina paired-end reads (*right*). (*E*) The eight-copy *MYC* region of LC2/ad represented by the optical mapping method. Optical maps with rearrangements and Chromosome 8 (reference) are represented in light blue and yellow-green, respectively. The orange arrow indicates the *MYC* gene. (*F*) Comparison of SVs of SK-BR-3 cells from PacBio data (red; previous report) with PromethION data (green). A total of 12,392 SVs were common between the two data sets (*upper* panel). Percentage of unique SVs overlapping with the other data set when the number of required reads supporting SVs decreased. Colors of the lines correspond to the colors of the regions in the upper Venn diagram (*lower* panel). (*G*) Comparison of genic SVs of SK-BR-3 cells from PacBio data with PromethION data in the same manner as *F*. (*H*) Large-scale translocations from the region around the *ERBB2* gene to Chromosome 8 detected by the PromethION data. The translocations corresponded to the previous report using PacBio data.

By using the long-read sequencing data, we attempted to detect structural aberrations. From the MinION/PromethION data set of LC2/ad, we successfully identified 12 reads directly overlapping the junction point of the *CCDC6-RET* fusion gene, which is the most important cancer driver mutation for this cell line (for details of the bioinformatics pipeline, see Methods) (Fig. 2A; Matsubara et al. 2012; Suzuki et al. 2013b). We further attempted to identify other large deletions. We identified a large deletion around the *CDKN2A* gene, which is a well-known tumor suppressor gene (The Cancer Genome Atlas Research Network 2014), in LC2/ad, A549, and PC-14 cells (Fig. 2B; Suzuki et al. 2014). By using the MinION/PromethION data sets in this study, we reconfirmed the deletion of this gene in the respective cells. In addition, we found that the precise junction point of each of the *CDKN2A* deletions differed between the cell types. Large deletions in other cancer-related genes are described in Supplemental Figure S4. We could also detect novel gene fusions by using the split alignment method (see Methods). We identified six novel rearrangements, which were further supported by the Illumina short reads. These genes were fused to *EFNA5-IKBKB*, *NELL1-CCSER1*, *EFNA5-POLB*, *ANKS1B-TRPM3*, and *ANKS1B-CEMIP2* in LC2/ad and *UTS2B-GRM4* in RERF-LC-KJ. In each of these cases, the long-read sequencing precisely identified the potential junction sites (Supplemental Table S3). We also examined the general sensitivity and specificity of the medium- or large-scale SV (≥2 kb) calls based on the long-read sequencing data (for more details, see Supplemental Fig. S5A). By using the PromethION data in LC2/ad, we found that the detection sensitivity depends on the sequencing depth (Fig. 2C). We also found that it was dependent on the type of SV contexts, having decreasing call accuracy for duplications, translocations, inversions, and deletions. At the 20× sequencing depth, 90% of the duplications were detected, which was higher than for the other SV types. Comparison of the detected SVs from PromethION with those from Illumina short reads is shown in Supplemental Figure S5B and Supplemental Table S4.

To attempt a more popular method of SV detection, we also conducted the genome-wide SV detection using NGMLR and Sniffles (Sedlazeck et al. 2018). From this approach, we identified SVs with ≥50 bp, including deletions, insertions, duplications, inversions, and translocations, using the PromethION data sets of LC2/ad. We compared the results between the long-read (PromethION, Sniffles) and short-read (Illumina, GenomonSV) approaches (Kataoka et al. 2016). We excluded insertions from the comparison because long reads are known to be superior to short reads for the detection of insertions, as previously reported (Nattestad et al. 2018). Of 17,092 SVs from the PromethION data set, 2236 SVs were also detected in the Illumina data set (Supplemental Fig. S5C–F). The SV total number and intersections between the long- and short-read data sets were similar to those in the previous report (Nattestad et al. 2018).

We further attempted to decipher, perhaps, the most difficult case, the rearrangement of the *MYC* gene, by taking advantage of long-read sequencing. We had previously identified possible copy-number aberrations in the *MYC* gene in LC2/ad (Suzuki et al. 2014). The amplification was estimated to extend a >8 Mb locus, having the *MYC* gene at its center. However, we failed to decipher its complete structure. Even with long-read sequencing, it was still difficult for us to completely reconstruct its structure, which included complex rearranged patterns, expanding to 8 Mb in Chromosome 8 at an estimated aneuploidy of eight (Fig. 2D). Particularly for the *MYC* region, we attempted to identify the correct structure by the optical mapping method, Bionano Saphyr. Even with Saphyr, the precise structure of the *MYC* region remained elusive, although the results from this analysis support the *MYC* amplification spanning the 8-Mb region with approximately eight copies (Fig. 2E).

### Comparison with PacBio long-read sequencing

To further assess the performance of PromethION to detect SVs from another perspective, we attempted to compare the results with those obtained from another long-read sequencer, PacBio, conducted by a different group (Nattestad et al. 2018). We selected this data set because this study is the most well-suited work of long-read cancer genome sequencing and the complete set of the basic data was publicly available. For the comparison, we conducted the whole-genome PromethION sequencing using the same cell line, SK-BR-3, which is a HER2-positive breast cancer cell line (Supplemental Fig. S6; Supplemental Table S5). We mapped the sequencing data to the human reference genome by using NGMLR and called the SVs by using Sniffles (Supplemental Fig. S7; Sedlazeck et al. 2018).

As for the obtained data sets, we compared the results between PacBio and PromethION. Most (80%) of the SVs called from the PromethION data were consistent with those called from the PacBio data (12,392 out of 15,408 SVs) (Fig. 2F; Supplemental Fig. S7). By further examining the cases of discrepancy, we attempted to characterize the possible causes. We found that the number of insertions called from the PacBio data was larger than that called from the PromethION data, whereas the number of duplications called from the PromethION data was larger. The other types of SVs, such as deletions, inversions, translocations, and inverted duplications, were the same between the two data sets (Supplemental Fig. S7). As for the SVs detected only from the PacBio data, ∼19% of the SVs could also be called from the PromethION data by lowering the threshold of the supporting reads. Conversely, as for the SVs detected only from the PromethION data, ∼28% of the SVs could be called from the PacBio data when a lower threshold of reads supporting SVs was used (Fig. 2F). Some of the discrepancy is thus from the different read coverage of the sequencings, which remain to be completed (see below for further analyses using clinical samples, where cancer cell contents are not 100%; thus, deeper sequencing is generally needed).

In particular, we focused on the SVs located in the gene loci. About 81% of these SVs overlapped between the PacBio data and the PromethION data (4806 out of 5934 SVs). When we lowered the threshold of the supporting reads, an increased number of consistent cases were detected (20% and 26% of the inconsistent cases of PacBio- and PromethION-called SVs, respectively; Fig. 2G). We further inspected the still inconsistent cases and determined that, in most of these cases, relatively small indels were found, for which it was difficult to decipher whether the PromethION or the PacBio results are correct. However, most of them appear to have no particular biological significance, being located within a deep intronic region or far upstream/downstream. Nevertheless, we detected several putatively biologically meaningful cases. For example, we detected a duplication spanning a genomic region of >10 kb in Chromosome 1 only from the PromethION data (Supplemental Fig. S7C). Both end junctions of the SVs resided in the gene regions: within the *CHTOP* and *KCNN3* genes. We detected 76 and 23 SVs only from PacBio or PromethION, respectively, when we counted SVs overlapping with coding regions, thus affecting their functions. These results indicated that neither the PacBio nor the PromethION platform is currently perfect; therefore, they should be used to complement each other.

We also attempted and failed to detect amplification around the region of the *ERBB2* gene in Chromosome 17 by either PromethION or PacBio. Similar to the case of the *MYC* gene, this region consisted of at least five tandemly arrayed translocations (Fig. 2H). Despite the significant improvements of the long-read sequencing and its analytical pipelines, careful manual inspections are still needed, particularly for targets of primary biological interest.

### Identification of complicated cancerous local genomic lesions

**Figure 3.**
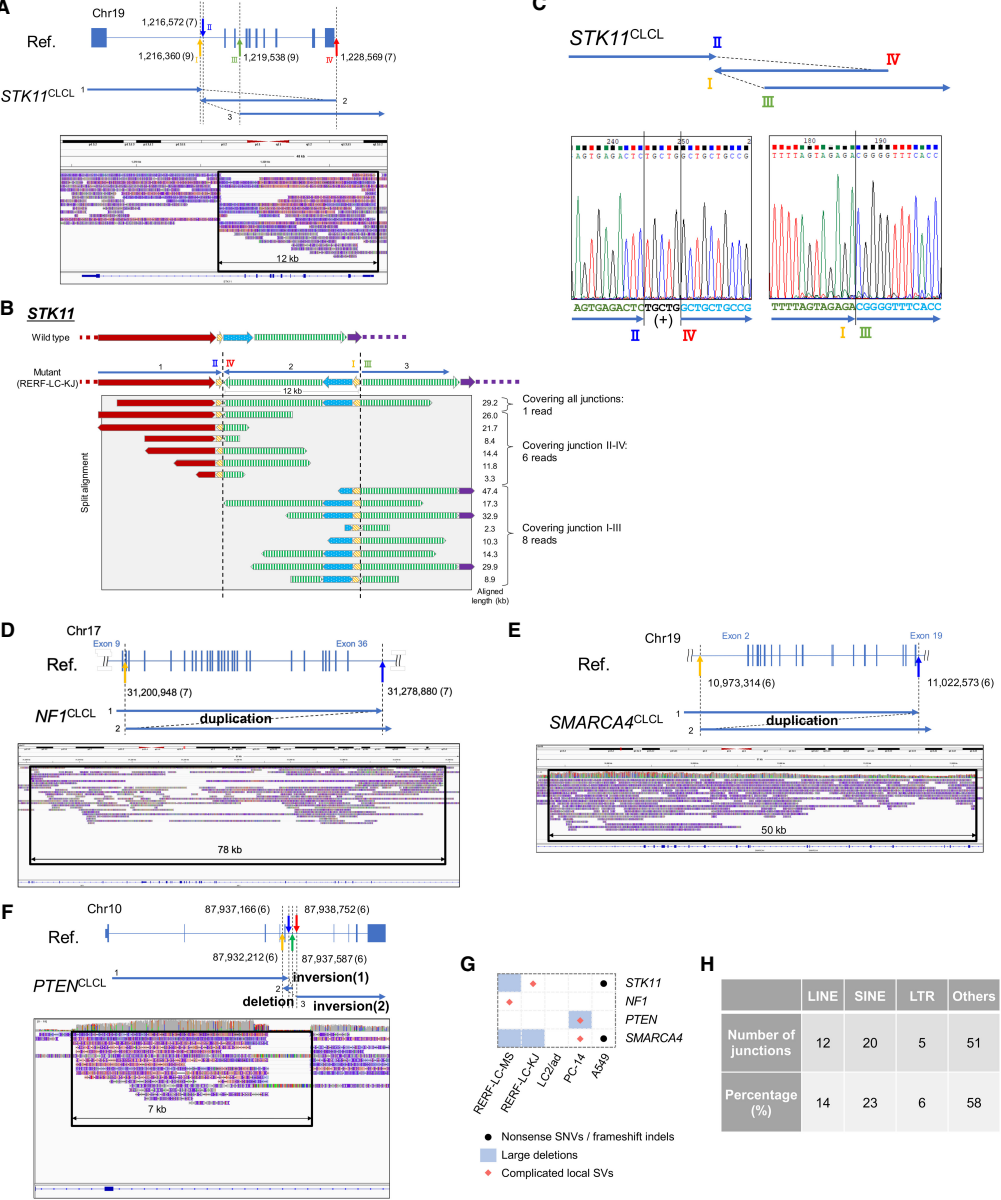
Identification and characterization of complicated local structural aberrations. (*A*) Structure of *STK11* in RERF-LC-KJ. The *STK11* CLCL was constructed by a combination of local inversions. We can trace the structure following the ordered arrows, and the junctions are indicated by colored arrows. The SV spanned 12 kb in the human reference genome. (*B*) Information on long reads for the complicated structure of the *STK11* CLCL. The SV structure and aligned reads of PromethION are shown in the *upper* and *lower* panels. Aligned length is represented in the margin. (*C*) The results of Sanger sequencing of junctions of *STK11* CLCL. The CLCL junctions of *STK11* in RERF-LC-KJ were validated by Sanger sequencing. The CLCL structure is shown in the *top* panel. The 5-bp insertion was observed in junction I/III. PCR and sequencing primers are shown in the Methods section. (*D*) Structure of the *NF1* in RERF-LC-MS. The structure of the SV was a tandem duplication between the junctions (indicated by a yellow arrow and a blue arrow). The SV spanned 78 kb in the reference genome. (*E*) Structure of the *SMARCA4* in PC-14. The structure of the SV was a tandem duplication of the junctions (indicated by a yellow arrow and a blue arrow). The SV spanned 50 kb in the reference genome. (*F*) Aberrant genomic structure of *PTEN* in PC-14. The structure of the SV was a combination of a local inversion and deletion. We can trace the SV structure following the ordered arrows, and the junctions are indicated by colored arrows. The SV spanned 7 kb in the reference genome. (*G*) Summary of mutation types of four cancer-related genes in five cell lines. (*H*) The number of complicated SV junctions in each category of genomic context.

During the attempts to identify the above structural aberrations of the established classes, we found a new type of local structural aberration (Fig. 3). These aberrations consisted of complex combinations of copy-number changes (duplications), inversions, and deletions. As it appears that these aberrations do not precisely belong to the above categories, we named them “cancerous local copy-number lesions” (CLCLs). As we describe below, we found it difficult to identify and characterize these CLCLs regarding their precise junctions based solely on short-read sequencing, although some suggestive data could occasionally be obtained.

The first example was found in the *STK11* gene locus. In our previous study of lung cancer whole-genome sequencing using Illumina, we noticed a possible local copy-number lesion in the *STK11* gene region in RERF-LC-KJ cells. The sequencing depth increased from the middle of intron 1 to the end of the gene (Suzuki et al. 2014). There were short-read split tags (for details, see Methods), suggesting that inversions may occur in this region. Despite the substantial number of sequencing reads mapped in this region, we could not reconstruct its precise structure.

We examined the long reads to decipher the aberration in the *STK11* gene locus (Fig. 3A). It revealed the aberration as follows: The first rearrangement occurred as an inversion starting from intron 1 (Chr 19: 1,216,572; breakpoint II) and jumping downstream from the gene (Chr 19: 1,228,569; breakpoint IV). The inverted sequence continued back to the middle of intron 1 (Chr 19: 1,216,360; breakpoint I), which was 212 bases upstream of the initial breakpoint II. Then, the sequence reverted back and jumped to intron 3 (Chr 19: 1,219,538; breakpoint III). The following sequence continued to the end of the gene locus. The detected junctions, breakpoints II/IV and I/III, were represented by seven and nine PromethION reads, respectively. One read completely covered the structure. The individual reads corresponding to this aberration are shown in Figure 3B (lower panel). When we re-examined the Illumina reads, the sequencing depth increased at two regions, between breakpoints I and II and between breakpoints III and IV (boxed region in the upper panel in Fig. 3A). We also looked for the short reads using the soft-clipped method. We found that it was difficult to detect breakpoint I/III by using the short-read split tags, partly because the junctions resided in the repetitive regions. To further address this issue, we conducted Sanger sequencing of the junction points (Fig. 3C). We finally identified the disordered part of the gene, which was sufficiently complicated so as not to be detected by short reads.

To more generally identify CLCLs with copy-number changes in other loci in all lung cancer cell lines, we constructed a new analytical bioinformatics pipeline (see Methods; Supplemental Fig. S8). Briefly, we used the information of the split alignments from the mapping results. We sorted the mapping information by the position of the reads and extracted the CLCL candidates. The associated reads were reassembled to reconstruct their structures. Thus, we successfully identified the following numbers of CLCLs with local duplications in the other cell lines as well: 14 in LC2/ad, one in A549, eight in RERF-LC-KJ, seven in RERF-LC-MS, and 12 in PC-14 (Table 2). CLCLs were found to occur even in key cancer genes, such as the *STK11*, *NF1*, *SMARCA4*, and *PTEN* genes. The aberrant structures varied, and most of them could not be easily detected by the conventional short-read-based approaches because of their complex structures and the size of the affected regions. A relatively simple one was that which was detected in the *NF1* gene in RERF-LC-MS cells (Fig. 3D). This was a tandem duplication of the region between intron 9 (Chr 17: 31,200,948) and the downstream region of the last exon 36 (Chr 17: 31,278,880; it was supported by seven reads at the junction). In another case, the structure of the *SMARCA4* CLCL showed a duplication from intron 1 (Chr 19: 10,973,314) to intron 20 (Chr 19: 11,022,573; supported by six reads at the junction) (Fig. 3E). A more complex case was found in the structure of *PTEN* in PC-14. This CLCL was found to be a combination of inversion and deletion (Fig. 3F; Supplemental Fig. S9A). The junctions of *PTEN* CLCL were validated by Sanger sequencing analysis (Supplemental Fig. S10). For these cases, once identified, remapping of the Illumina short reads to the identified junctions could validate the reconstructed structure. Indeed, the presence of these mutations was partly suspected in a previous study (Suzuki et al. 2014). However, their precise structures remained elusive before this study by conventional aberration detection based on the short reads (Fig. 3G).

**Table 2.**
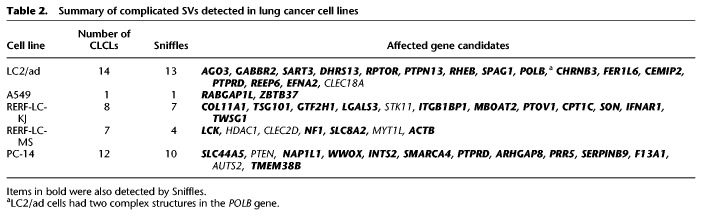
Summary of complicated SVs detected in lung cancer cell lines

More generally, we also examined the genomic context of these CLCLs. In total, 67% (28/42) of the SVs had at least one junction overlapping with a long interspersed nuclear element (LINE), short interspersed nuclear element (SINE), or long terminal repeat (LTR), and 14%, 23%, and 6% (12/88, 20/88, and 5/88, respectively) of the junctions of the SVs were in a LINE, SINE, or LTR, respectively (Fig. 3H). It is possible that their repetitive regions may hamper the precise identification of SVs by short-read sequencing.

### Aberrant transcriptional events associated with CLCLs

**Figure 4.**
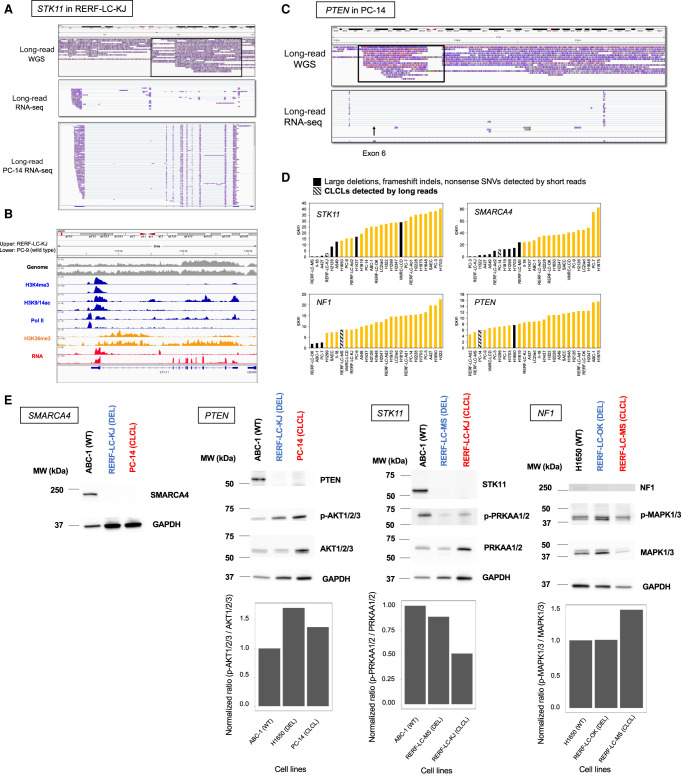
Aberrant transcriptional events caused by complicated local structural aberrations. (*A*) Structures of *STK11* transcripts in RERF-LC-KJ. Sequencing tags of whole-genome sequencing (PromethION) and full-length RNA-seq (MinION) were visualized by IGV. PC-14 RNA-seq was also shown as a wild-type control. (*B*) Multilayered statuses in the *STK11* region. Patterns of whole-genome sequencing, ChIP-seq, and RNA-seq tags of short-read data were visualized by IGV. The status of RERF-LC-KJ and PC-9 (control) is shown. (*C*) Structures of *PTEN* transcripts in PC-14. Sequencing tags of whole-genome sequencing (PromethION) and full-length RNA-seq (MinION) were visualized by IGV. The transcripts indicated that exon 6 was skipped (black arrow). (*D*) Expression levels of *STK11*, *NF1*, *SMARCA4*, and *PTEN* in 26 lung cancer cell lines. Cell lines with deleterious mutations, such as large deletions, frameshift indels, and nonsense SNVs, are shown in black. Cell lines with complicated SVs are also shown in black with a diagonal line. (*E*) Western blotting of genes affected by complicated SVs and their downstream targets. (MW) Molecular weight; (WT) wild type; (DEL) large deletion; (CLCL) complicated local SV focused on in this study. Bar charts indicate the normalized ratio of density of phosphorylated proteins and total proteins. Each protein is downstream from proteins encoded by the genes.

After the identification of complicated patterns of aberrations in several key genes of many cell lines, we wondered what transcriptional or epigenomic consequences they have. To characterize how the aberrations are reflected in the transcriptome, we newly generated and analyzed full-length cDNA sequencing data using MinION. We also used the previous Illumina short-read RNA-seq and ChIP-seq data. In RERF-LC-KJ cells, short-read sequences indicated that the *STK11* transcript is abnormally spliced at intron 1 and that transcription jumped just before the aberrant genomic structure (Suzuki et al. 2014). MinION reads representing the full-length transcripts further specified the precise splice pattern and the transcription termination sites (Fig. 4A). For almost all the transcripts, the first splicing occurred at an abnormal position (from Chr 19: 1,216,268) and transcription occurred according to the aberrant structure (RNA-seq reads covered breakpoints II–IV from Chr 19: 1,216,572 to Chr 19: 1,228,569). Such an aberrant transcription pattern was not observed in PC-14 cells, where *STK11* is the wild-type gene (Fig. 4A, lower panel).

We examined the epigenome marks in the regions surrounding the complicated local SV as represented by the ChIP-seq of H3K4me3, H3K9/14ac, and RNA polymerase II. We found that the chromatin normally formed the active structure at the promoter regions and that transcription was initiated normally at the correct position regardless of whether the cell line harbored the mutant or wild-type *STK11* locus (Fig. 4B). However, in only the RERF-LC-KJ cells harboring the CLCL, the H3K36me3 mark disappeared in the middle of intron 1, indicating that transcriptional elongation should be disrupted exactly where the SV started. Illumina RNA-seq data also supported that the RNAs were abnormally spliced in the middle of intron 1 and transcribed according to the aberrant genomic structure. The expression levels of these aberrant transcripts were measured as 2.8 reads per kilobase of exon per million mapped fragments. No normal transcripts were detected. However, the aberrant transcripts retained a substantial expression level, although somewhat lower than that of the wild type.

We conducted a similar analysis for the CLCLs. For the *PTEN* gene in PC-14 (Fig. 4C), the CLCL resided at exon 6 (RefSeq ID: NM_001304717). As a result, this exon was completely skipped from the transcripts of *PTEN*. Accordingly, the resulting transcript should be frame-shifted and thus would be likely to lead to functional loss of the *PTEN* gene. We also examined the RNA expression levels in the *STK11*, *NF1*, *SMARCA4*, and *PTEN* genes harboring CLCLs based on the Illumina RNA-seq data. The results indicated that CLCLs are generally likely to result in reduced gene expression levels (Fig. 4D). Nevertheless, in some cases, gene expression levels remained significant, such as the *NF1* transcripts in RERF-LC-MS cells and the *PTEN* transcripts in PC-14 cells.

To address the biological significance of the CLCLs, we examined how the SV-affected locus invokes changes in protein expression levels and their related signaling pathways. We conducted western blotting analysis. As expected, we found that the proteins of STK11, NF1, SMARCA4, and PTEN were completely lost in cells harboring CLCLs in these genes (Fig. 4E). We further examined the activation status of the downstream proteins. The expected disruptions of the pathways were observed for all the examined cases. PTEN suppresses the phosphorylation of AKT1/2/3, and phosphorylated AKT1/2/3 (phospho-AKT1/2/3) consequently activates the mTOR signaling pathway (Carracedo et al. 2008). Aberrant up-regulation of phospho-AKT1/2/3 was observed, reflecting the functional loss of PTEN in PC-14 cells (PTEN-CLCL). *PRKAA1/2* is a gene that plays an important role in maintaining cellular homeostasis, and the phosphorylation of the PRKAA1/2 protein at its alpha subunit is activated by STK11 (Mihaylova and Shaw 2012). Its activation is impaired in RERF-LC-KJ cells (STK11-CLCL). The *NF1* gene is a negative regulator of RAS oncoprotein with tumor suppressor function (Wolman et al. 2014). Phospho-MAPK1/3, which is downstream from the RAS signaling pathway (De Luca et al. 2012), was aberrantly up-regulated in RERF-LC-MS cells (NF1-CLCL). Despite the clear protein losses of the corresponding genes in all cases, either by conventional aberrations or by complicated SVs, their consequences varied to a certain extent depending on the case. For example, even though the STK11 protein similarly disappeared in both RERF-LC-MS cells (STK11-DEL) and RERF-LC-KJ cells (STK11-CLCL), the enhanced ratio of phospho-PRKAA1/2 was lower in the RERF-LC-KJ cells. The effects of NF1 in RERF-LC-OK (NF1-DEL) were almost undetectable, whereas the effects were significant in RERF-LC-MS cells (NF1-CLCL). Other pathways may sometimes complement the loss of the key protein. Future comprehensive work using large data sets, such as DepMap (Tsherniak et al. 2017), would address this issue.

### Detection of CLCLs in clinical samples

**Figure 5.**
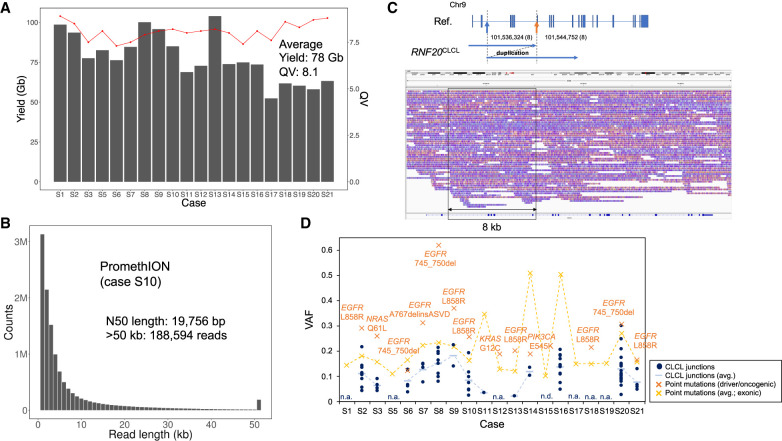
Analysis of lung cancer clinical samples. (*A*) Sequencing yields and mean quality values of the data sets from PromethION. (*B*) Raw read length distribution of PromethION sequencing in case S10 (two flow cells; 28×). The N50 length and the number of reads >50 kb are shown in the *inset*. (*C*) The CLCL of *RNF20* in case S8. An aberrant structure of *RNF20* is shown in the *upper* panel. The structure was a tandem duplication between the junctions (blue arrow and yellow arrow). IGV visualization is represented in the *lower* panel. The aberrant structure (∼8 kb) is indicated in the box. (*D*) Variant allele frequencies of CLCL junctions of 20 clinical samples. Variant allele frequencies (VAFs) of CLCL junctions (blue) are shown in comparison with those of point mutations (yellow). VAFs were calculated using Illumina short-read data by Genomon and GenomonSV (VAF > 0.01). Of the 95 CLCL junctions, 79 junctions were also detected in GenomonSV. Driver/oncogenic mutations (known mutations in *EGFR*, *KRAS*, *NRAS*, and *PIK3CA*) were detected in 13 cases, and their VAFs are presented in orange. No CLCLs were detected in six cases (S1, S5, S12, S17, S18, and S19). (n.a.) Not available; (n.d.) not detected by GenomonSV; thus, the VAF should be considerably low. The estimated VAFs of SNVs and SVs are simultaneously plotted on the same planner for the purpose of comparison, although the accuracy for the VAFs of SVs is not fully validated.

To examine CLCLs in clinical cases of lung adenocarcinoma, we conducted similar PromethION whole-genome sequencing for the surgical specimens of 20 Japanese patients with lung adenocarcinoma (Table 3; Supplemental Fig. S11). The detected driver mutations for each patient are shown in Supplemental Table S6. For these cases, we generated >78 Gb of sequences on average for each case (>25× depth) (Table 3A; Fig. 5A). For all cases, we also sequenced normal counterparts to eliminate possible normal variations and dubious SVs derived from the mapping errors (Table 3B). To show a representative case (case S10), the maximum and N50 lengths of the reads were 584,753 and 19,756 bp, respectively (Fig. 5B). Statistics for the other cases are shown in Figure 5A and Table 3.

**Table 3.**
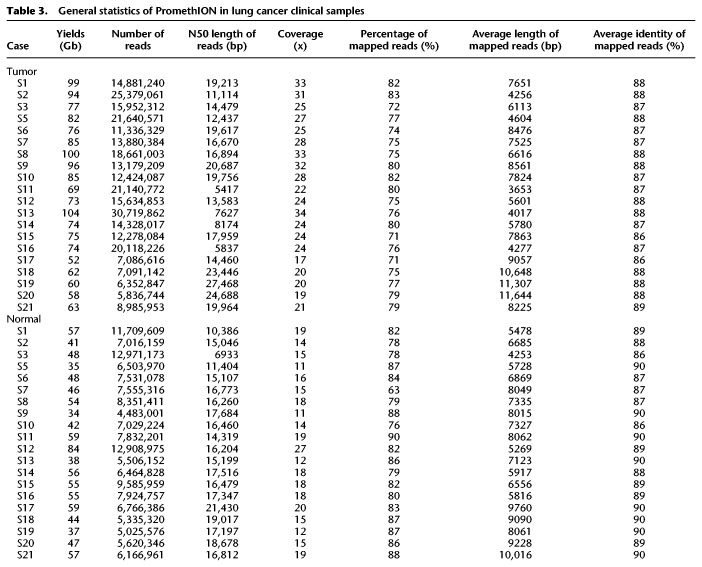
General statistics of PromethION in lung cancer clinical samples

We also examined the presence of these CLCLs in clinical samples. We found that 14 of the 20 specimens harbored at least one such CLCL (Table 4). Again, several key cancer genes were included. For example, we identified an *RNF20* CLCL in case S8. This patient is a female who has been shown to have an *EGFR* exon 19 deletion as a driver mutation. However, the other cancerous mutations remained elusive. In this case, the CLCL of the *RNF20* gene occurred as a tandem duplication between intron 2 (Chr 9: 101,536,324) and intron 6 (Chr 9: 101,544,752) (Fig. 5C; Supplemental Fig. S9D), which is very likely to lead to the functional loss of this gene. The *RNF20* gene encodes an E3 ubiquitin ligase with tumor suppressor function, and it is frequently mutated, particularly in lung cancer (Sethi et al. 2018). In the other cases, the indications obtained for the molecular etiology underlying the carcinogenesis of the patients are summarized in Supplemental Table S6. In total, 76 CLCLs were detected, which included 14 CLCLs with complicated combinations of tandem duplication and other types of SVs. Further scaling of the long-read sequencing would be needed to more precisely identify the frequencies of the CLCLs and the genes preferentially harboring such SVs.

**Table 4.**
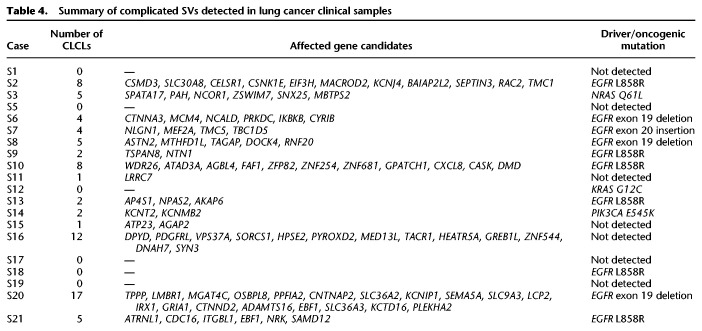
Summary of complicated SVs detected in lung cancer clinical samples

We were also concerned whether the tumor purity and intra-tumor heterogeneity may affect the detection of CLCLs. To address this issue, using the short-read data, we calculated variant allele frequencies (VAF) in the junctions of the CLCLs by using GenomonSV and compared them with those of point mutations including known driver events. The VAFs of driver mutations have been proposed to represent the tumor contents in many previous studies (Fig. 5D; Roth et al. 2014; Smits et al. 2014; Andor et al. 2016). Neither CLCLs nor any driver events were detected in four cases. We suspect that the tumor content of the specimens was considerably low or that cancer cells were highly heterogeneous in these cases. For six cases, there was no CLCL detected. For the other cases, in which CLCLs were detected, the VAFs of driver/oncogenic mutations ranged from 0.12 to 0.62, indicating that the tumor purity was expected to be ≥20%. The VAFs of overall point mutations were also consistent. For these cases, the VAFs of the CLCLs ranged from 0.02 to 0.30. The VAFs of CLCL junctions were considered to be highly correlated with those of driver mutations or other point mutations, as the VAFs of CLCL junctions are supposed to be lower than the VAFs of SNVs because the current criteria to evaluate the VAFs of CLCLs are less straightforward than the case of SNVs. Nevertheless, we could detect at least major CLCLs such as driver mutations for each specimen. Because the primary goal of this study was to identify CLCLs of possible cancer-causing SVs, for which the VAF levels are similar to those of driver mutations, we considered that the current sequencing depth is sufficient for this purpose. Additional sequencing depths would further detect additional CLCLs, which occur in a minor population of cancer cells, and thus may play a role in the heterogeneity of cancer cells or in cancer evolution.

## Discussion

In this study, we have described the identification and characterization of complicated structural aberrations in lung cancer genomes by using PromethION. We could identify the precise junctions of chromosomal rearrangements and large-scale deletions relatively easily. For example, the junction points of the *CDKN2A* gene were precisely detected (Fig. 2B). In most cases, the proximal genes were simultaneously deleted. In LC2/ad, the deletion spanned from the *MIR31HG* and *MTAP* gene loci to the *DMRTA1* gene locus (supported by nine reads). In A549 cells, the deletion started from the *MTAP* locus and reached the *CDKN2B* gene locus (supported by 15 reads). In PC-14, 22 genes were deleted in addition to the *CDKN2A* gene (supported by eight reads). Several studies have reported that *CDKN2A* codeleted genes are involved in the hidden molecular features of cancers. The *MTAP* gene encodes 5-methylthioadenosine phosphorylase, which is associated with the purine and methionine salvage pathways, located in the region adjacent to the *CDKN2A* gene and frequently codeleted in cancers. *MTAP*-deficient cancers are known to acquire vulnerability to arginine methyltransferase PRMT5 depletion, which may be a novel target of an anticancer drug (Kryukov et al. 2016; Marjon et al. 2016; Mavrakis et al. 2016). It is important to determine the precise junction by the long-read approach to completely understand the genes or regions that are affected by these genomic aberrations.

We also identified the 8-Mb amplification of the *MYC* gene locus in LC2/ad cells. We attempted to further characterize it and found that even the latest long-read sequencing technologies could not characterize the precise mutation pattern in this locus. Eight megabases may have been considerably large and the internal rearrangement may have been highly complicated, although this is the only region where we could not reassemble the structure. We found no sequences suggesting aberrations occurring within the internal region of the *MYC* gene locus itself. Outside of the *MYC* gene, at least four breakpoints were detected by nanopore reads, which were further confirmed by the Illumina short reads (Fig. 2D). There may be unique selective pressure exerted on this gene specifically, retaining the gene's function itself intact while at the same time enhancing its expression.

We identified a unique and complicated aberration pattern with local copy-number changes (CLCLs). We found that these CLCLs exist even in pivotal cancer-related genes, such as the *STK11*, *NF1*, *SMARCA4*, and *PTEN* genes. Recent studies have reported that immune checkpoint inhibitors are less effective for lung cancers with *STK11* mutations (Rizvi et al. 2018; Skoulidis et al. 2018). Therefore, the therapeutic strategy for each patient would differ depending on whether or not there is a mutation in the *STK11* gene. Additionally, CLCLs were identified in the *PTPN13*, *RPTOR*, and *RHEB* genes in LC2/ad cells (Table 2). *PTPN13* encodes a protein tyrosine phosphatase, whereas the *RPTOR* and *RHEB* genes are members of the MTOR signaling pathway. The functional loss of these genes could be related to tumorigenesis and malignancy of the cancer, although further studies will be needed to clarify the relationship between those aberrations and the molecular etiology of cancers in more detail.

We could also analyze the causes and consequences of genomic CLCLs. In total, 67% of the SVs had at least one junction in LINE, SINE, and LTR regions, suggesting that transposable elements were likely to contribute to the formation of aberrant structures. By using epigenome and transcriptome data, we also showed that complicated local SVs led to the formation of abnormal transcripts and functional loss of their encoded proteins in most cases.

We further conducted long-read sequencing of clinical samples. We successfully showed that CLCLs occur in the in vivo genomes of patients with lung adenocarcinoma. In 14 cases, at least one CLCL was detected in the genes with important functions, providing a complementary therapeutic indication for the patients. As expected, the detected CLCLs in each patient did not overlap with those from the other cases because most of mutations are not generally recurrent, except for some hotspot mutations. In clinical samples, we also faced difficulties of mutation detection, which is caused by low tumor purity and high heterogeneity of the tumor cells. Conventional short-read sequencing tends to cause erroneous detection, although it is easy to obtain more data than those from long-read sequencers, which need large amounts of intact DNA samples for whole-genome sequencing. Nevertheless, we attempted to reanalyze short-read sequencing data from clinical samples that were previously published (Suzuki et al. 2013a; The Cancer Genome Atlas Research Network 2014), particularly focusing on detecting CLCLs in cancer-related genes, such as the driver genes of lung adenocarcinoma (Supplemental Fig. S12). For example, in the case of TCGA-49-4512 (female, nonsmoker), we identified a potential complicated SV in the kinase domain of the *EGFR* gene. This duplication was previously reported (Gallant et al. 2015) and might cause aberrant activation of EGFR, thus serving as a driver mutation of this case. This patient's therapeutic target should be addressed by EGFR inhibitors such as afatinib (Gallant et al. 2015). We believe that it is important to subject those cases to further detailed long-read sequencing. We tentatively call these complicated local SV CLCLs as a new category of SVs for further extensive long-read sequencing analysis. Future studies should reveal how CLCLs occurring in cancer genomes play a role in the phenotypic features of a given cancer, including its response to anticancer drugs.

Lastly, we found that visualization of the detected mutation is also important. The so-called “genome graph” data structure should play an indispensable role in representing the diverse nature of cancer genomes and thus further enhance the accuracy of future genome analyses (Garrison et al. 2018; Rakocevic et al. 2019). Recently, a tool for visualizing SVs based on a genome graph has been developed (Yokoyama et al. 2019). For the graph genome approach, eventually all the various SVs should be collectively represented. To this end, distinct approaches may be needed depending on the variant types including CLCLs. This is the first study that has used PromethION sequencing for cancer genomics. Obviously, further improvements in the sequencing method itself, coupled with refinements of the computational tools, are needed to achieve for more accurate detection of SVs in cancer genomes. Indeed, this study may have raised more questions than answers. In that sense, this is only the first study paving the way toward a more comprehensive understanding of the complicated genomic aberrations of cancers and further in-depth study of their biology.

## Methods

### Cell lines and clinical samples

The lung adenocarcinoma cell lines LC2/ad, A549, RERF-LC-KJ, RERF-LC-MS, and PC-14 were cultured as previously described (Suzuki et al. 2014). SK-BR-3 (ATCC HTB-30) was cultured using McCoy's 5A Medium (30-2007 ATCC). Cell pellets were washed with cold PBS and cryopreserved.

Clinical samples were obtained with the appropriate informed consent at the National Cancer Center Japan. Surgical specimens from 21 patients were pathologically checked, and case S4 was removed because of low tumor content (Supplemental Fig. S11; Supplemental Table S6). All 20 patients were diagnosed as having primary lung cancer, including 15 adenocarcinomas, two squamous cell carcinomas, one pleomorphic carcinoma, one LCNEC, and one large-cell carcinoma. Fresh-frozen surgical specimens were used to extract genomic DNA (gDNA) and total RNA as described below.

### Whole-genome sequencing using MinION

High-molecular-weight (HMW) gDNA was extracted from the lung cancer cell lines LC2/ad and A549 with the smart DNA prep(a) kit (Analytikjena). In the case of LC2/ad, WGS data were produced from 1D sequencing (SQK-LSK108), 1D^2^ sequencing (SQK-LSK308), and rapid sequencing (RAD003); in the case of A549, WGS data were produced from only 1D^2^ sequencing. Library preparation was conducted according to the manufacturer's instructions (also see Supplemental Methods).

### Whole-genome sequencing using PromethION

The HMW gDNA extraction method for LC2/ad and RERF-LC-MS was the same as that for the MinION sequencing. From RERF-LC-KJ, PC-14, and lung adenocarcinoma clinical samples, HMW gDNA was extracted with the MagAttract HMW DNA kit (Qiagen). Library preparation for 1D sequencing (SQK-LSK109) and sequencing using PromethION were conducted according to the manufacturer's instructions (also see Supplemental Methods).

### Computational analysis of long-read sequencing data

MinION fast5 data were base-called using albacore 2.0.2 and converted to FASTQ files. PromethION fast5 data were base-called using guppy and converted to FASTQ files. Our MinION and PromethION data sets were mapped to the human reference genome, hg38, using minimap2 (with the “-ax map-ont” option, 2.9-r720 version). MinION 1D^2^ sequencing outputs two types of FASTQ file, 1D and 1D^2^. 1D means that reads were generated using single-strand information. 1D^2^ reads integrate the double-strand information. There were some overlapping reads between 1D files and 1D^2^ files. Therefore, reads used as 1D^2^ were removed from the 1D files. In addition, there were some overlapping reads in the 1D^2^ files. These reads were removed from the 1D^2^ files and used as 1D reads.

### SNV calling using Illumina short reads

Whole-genome short-read sequences were mapped to the human reference genome (hg38) using BWA-MEM (version 0.7.15) (Li 2013). After mapping, sorted BAM files were created, and PCR duplicates were marked by SAMtools (Li et al. 2009). For the detection of SNVs of LC2/ad cells, we used GATK HaplotypeCaller (version 4.0.12.0) (Poplin et al. 2018) with base quality score recalibration and valiant quality score recalibration, and then we annotated SNPs by ANNOVAR using dbSNPs (Wang et al. 2010).

### SNV calling using PromethION long reads

SNVs were called from the PromethION data set of LC2/ad using Nanopolish (version 0.11.1) assuming the ploidy of two in the genome of Chromosome 22. For SNV calling, we annotated SNPs in the same fashion as in the Illumina analyses. For analysis of the known SNV, we added a “-d 10” option to the above command because of the lower sequence depth than the default parameter of 20.

### Full-length transcriptome sequencing using MinION

Full-length transcriptome analysis using MinION was performed as previously described (Seki et al. 2019). RNA was extracted from lung cancer cell lines by using the RNeasy mini kit (Qiagen). The extracted RNA was converted to cDNA by using a SMART-Seq v4 ultra low-input RNA kit (Takara). Then, we used cDNA as input for 1D^2^ MinION sequencing. Sequencing data were mapped to the human reference genome (UCSC hg38) using minimap2 (with the “-ax splice” option, 2.9-r720 version) and converted to a sorted BAM file using SAMtools.

### Analysis of SVs of SK-BR-3 cells

Sequencing reads by PromethION were mapped to the human reference genome, UCSC hg38, using NGMLR (with the “-x ont” option, 0.2.7 version). Then, SVs were called by Sniffles (version 1.0.11). A VCF file of SVs from PacBio data was downloaded from the site of a laboratory of investigators (Nattestad et al. 2018). The file was generated based on the reference genome, hg19. We lifted over positions in the file to hg38 and then compared the results with PromethION data. Figure 2H was made using SplitThreader (https://github.com/MariaNattestad/SplitThreader), and Supplemental Figure S7C was made using Ribbon (Nattestad et al. 2020).

### Detection of driver mutations for cell lines in long-read data

For the point mutations, we directly explored the known positions of the mutations using IGV. For the driver mutations of LC2/ad cells, the *CCDC6-RET* fusion gene, we explored the reads split-aligned to both *RET* and *CCDC6* genes in the reference genome and extracted the alignments with SAM format. Then we extracted the information of split alignment (chromosome, position of reference, read strand, and position of read) from the file and sorted the information by the position of reads. We filtered out reads with low mapping quality (MAPQ < 30) and counted the number of supporting reads.

### Detection of CLCLs from long-read data

To detect CLCLs, we used our method using the information of split read alignments. The scripts are available as Supplemental Code. The representative cases are visualized in the database DBKERO (https://kero.hgc.jp/) (Supplemental Fig. S13). First, we mapped sequencing data (FASTQ) to the human reference genome, hg38. Then we clustered split reads with IDs of reads from output files of mapping (BAM). We filtered out reads with multiple hits (flag: 256). Next, we extracted the information of split alignments (chromosome, position of reference, read strand, and position of read) from the file and sorted the information by the position of reads. We filtered out reads with low MAPQ (MAPQ < 30) from the data set. We extracted junction candidates of translocations, tandem duplications, and inversions considering the position of reads (removing junctions with large differences in read position, >300 bp), annotated the junctions of RefSeq genes, and merged the junctions <50 bp from the junctions of candidates. The threshold for the number of reads supporting the junctions was five. We generated sequence data with depth of at least 10× for all samples and could call heterozygous variants strictly at the threshold. We removed junctions with <2000 bp between the junctions because we aimed to detect medium-scale or large-scale SVs. CLCLs were detected when the gene or proximal genes contained at least two junction points. Finally, we checked and reconstructed the structures of translocations, tandem duplications, and CLCLs. To detect deletions, we used the information of split alignments and CIGAR strings in SAM format files. By using information of split alignments, we performed analysis in the same fashion as for translocations, tandem duplications, and inversions. We extracted the CIGAR strings from the BAM file with the filtering out of reads with multiple hits and low MAPQ. Then we integrated the results from analyses using information of split alignments and CIGAR strings, and then detected deletions of >2000 bp. We removed redundant SVs among samples. For clinical samples, we set SVs from normal samples as a control panel and removed the SVs from tumor samples whose support tags existed in normal samples. We also conducted analysis using Sniffles (version 1.0.11) with the “-s 5 -q 30” option for the cell lines.

### Optical mapping using the Saphyr system

Optical mapping analysis using the Saphyr system (Bionano Genomics) was performed for LC2/ad cells. Briefly, HMW DNAs were isolated from frozen cells by using a Bionano prep kit (Bionano Genomics) and measured by Qubit BR assay (Thermo Fisher Scientific). The extracted DNAs were fluorescently labeled with DLE-1 using a Bionano DLS kit (Bionano Genomics). Data were collected on the Saphyr instrument (Bionano Genomics). The figures were created by Bionano Access (version 1.3.0, Bionano Genomics).

### Sanger sequencing for validation of the CLCL junctions

Sanger sequencing was performed for two junctions of *STK11* CLCL in RERF-LC-KJ and two junctions of *PTEN* CLCL in PC-14 (Supplemental Methods). The chromatogram and sequence files used in the figures are provided as Supplemental Files.

### Western blotting

We performed western blotting as described previously to quantify proteins from genes with aberrant genomic structures (Supplemental Methods; Ohashi et al. 2015).

### Whole-genome short-read sequencing of clinical samples

gDNA was extracted from surgical specimens by using the MagAttract HMW DNA kit (Qiagen). Whole-genome sequencing libraries were constructed using the TruSeq nano DNA library prep kit (Illumina) and sequenced using NovaSeq, in accordance with the manufacturer's instructions. In summary, 100 ng of gDNA was used for library preparation as input, and DNA fragmentation, end repair, adenylation of the 3′ end, adapter ligation, and condensation of DNA fragments were conducted. We performed DNA fragmentation using Covaris and used a protocol for an insert size of 350 bp. After preparing the library, we denatured the library with NaOH and then started the NovaSeq run.

### Analysis of short-read sequencing data

For transcriptome and epigenome analyses of the cell lines, we used RNA-seq and ChIP-seq data that were previously obtained (DRA001846 and DRA001860) (Suzuki et al. 2014) and mapped to the reference genome hg19. IGV was used for visualization.

To detect SV junctions, GenomonSV (version 2.6.1) was used with paired-end read sequencing data as listed: five whole-genome data sets from cancer cell lines (DRA001859) (Suzuki et al. 2014) and 20 whole-genome data sets from Japanese patients with lung cancer. After conducting Genomon (version 2.6.1; https://genomon.readthedocs.io/ja/latest/; reference: hg19), GenomonSV filt was performed with the option “‐‐min_junc_num 1.” For clinical samples harboring matched normal data, we also set the option “‐‐matched_control_bam.” We merged SVs allowing 100-bp margins and removed redundancies among samples. For Supplemental Figures S5, B and C, and S12, the detailed methods were shown in the Supplemental Methods.

## Data access

The sequencing data of cell lines generated in this study have been submitted to the DNA Data Bank of Japan (DDBJ; https://www.ddbj.nig.ac.jp/index-e.html) under accession numbers DRA007423 (DRX143541, DRX143542, DRX143543, DRX143544), DRA007941, DRA008154, and DRA008295). The data from the cell lines have also been submitted to DBKERO (Suzuki et al. 2018; https://kero.hgc.jp). The sequencing data of the clinical samples have been submitted to the Japanese Genotype-phenotype Archive (JGA; http://trace.ddbj.nig.ac.jp/jga), which is hosted by the National Bioscience Database Center (NBDC) and DDBJ, under accession numbers JGAS00000000065 (JGAD00000000252 and JGAD00000000253).

## Competing interest statement

The authors declare no competing interests.

## Supplementary Material

Supplemental Material

## References

[GR261941SAKC1] Andor N, Graham TA, Jansen M, Xia LC, Aktipis CA, Petritsch C, Ji HP, Maley CC. 2016 Pan-cancer analysis of the extent and consequences of intra-tumor heterogeneity. Nat Med 22: 105–113. 10.1038/nm.3984 26618723 PMC4830693

[GR261941SAKC2] Ardui S, Ameur A, Vermeesch JR, Hestand MS. 2018 Single molecule real-time (SMRT) sequencing comes of age: applications and utilities for medical diagnostics. Nucleic Acids Res 46: 2159–2168. 10.1093/nar/gky066 29401301 PMC5861413

[GR261941SAKC3] The Cancer Genome Atlas Research Network. 2014 Comprehensive molecular profiling of lung adenocarcinoma. Nature 511: 543–550. 10.1038/nature13385 25079552 PMC4231481

[GR261941SAKC4] The Cancer Genome Atlas Research Network, Weinstein JN, Collisson EA, Mills GB, Shaw KRM, Ozenberger BA, Ellrott K, Shmulevich I, Sander C, Stuart JM. 2013 The Cancer Genome Atlas Pan-Cancer analysis project. Nat Genet 45: 1113–1120. 10.1038/ng.2764 24071849 PMC3919969

[GR261941SAKC5] Carracedo A, Salmena L, Pandolfi PP. 2008 SnapShot: PTEN signaling pathways. Cell 133: 550–550.e1. 10.1016/j.cell.2008.04.023 18455993

[GR261941SAKC6] Cavelier L, Ameur A, Häggqvist S, Höijer I, Cahill N, Olsson-Strömberg U, Hermanson M. 2015 Clonal distribution of *BCR-ABL1* mutations and splice isoforms by single-molecule long-read RNA sequencing. BMC Cancer 15: 45 10.1186/s12885-015-1046-y 25880391 PMC4335374

[GR261941SAKC7] Davis CF, Ricketts CJ, Wang M, Yang L, Cherniack AD, Shen H, Buhay C, Kang H, Kim SC, Fahey CC, 2014 The somatic genomic landscape of chromophobe renal cell carcinoma. Cancer Cell 26: 319–330. 10.1016/j.ccr.2014.07.014 25155756 PMC4160352

[GR261941SAKC8] De Luca A, Maiello MR, D'Alessio A, Pergameno M, Normanno N. 2012 The RAS/RAF/MEK/ERK and the PI3K/AKT signalling pathways: role in cancer pathogenesis and implications for therapeutic approaches. Expert Opin Ther Targets 16: S17–S27. 10.1517/14728222.2011.639361 22443084

[GR261941SAKC9] Gallant JN, Sheehan JH, Shaver TM, Bailey M, Lipson D, Chandramohan R, Brewer MR, York SJ, Kris MG, Pietenpol JA, 2015 *EGFR* kinase domain duplication (*EGFR*-KDD) is a novel oncogenic driver in lung cancer that is clinically responsive to afatinib. Cancer Discov 5: 1155–1163. 10.1158/2159-8290.CD-15-0654 26286086 PMC4631701

[GR261941SAKC10] Garrison E, Sirén J, Novak AM, Hickey G, Eizenga JM, Dawson ET, Jones W, Garg S, Markello C, Lin MF, 2018 Variation graph toolkit improves read mapping by representing genetic variation in the reference. Nat Biotechnol 36: 875–879. 10.1038/nbt.4227 30125266 PMC6126949

[GR261941SAKC11] Guan P, Sung WK. 2016 Structural variation detection using next-generation sequencing data: a comparative technical review. Methods 102: 36–49. 10.1016/j.ymeth.2016.01.020 26845461

[GR261941SAKC12] Imielinski M, Berger AH, Hammerman PS, Hernandez B, Pugh TJ, Hodis E, Cho J, Suh J, Capelletti M, Sivachenko A, 2012 Mapping the hallmarks of lung adenocarcinoma with massively parallel sequencing. Cell 150: 1107–1120. 10.1016/j.cell.2012.08.029 22980975 PMC3557932

[GR261941SAKC13] The International Cancer Genome Consortium. 2010 International network of cancer genome projects. Nature 464: 993–998. 10.1038/nature08987 20393554 PMC2902243

[GR261941SAKC14] Ishiura H, Doi K, Mitsui J, Yoshimura J, Matsukawa MK, Fujiyama A, Toyoshima Y, Kakita A, Takahashi H, Suzuki Y, 2018 Expansions of intronic TTTCA and TTTTA repeats in benign adult familial myoclonic epilepsy. Nat Genet 50: 581–590. 10.1038/s41588-018-0067-2 29507423

[GR261941SAKC15] Jain M, Koren S, Miga KH, Quick J, Rand AC, Sasani TA, Tyson JR, Beggs AD, Dilthey AT, Fiddes IT, 2018 Nanopore sequencing and assembly of a human genome with ultra-long reads. Nat Biotechnol 36: 338–345. 10.1038/nbt.4060 29431738 PMC5889714

[GR261941SAKC16] Kataoka K, Shiraishi Y, Takeda Y, Sakata S, Matsumoto M, Nagano S, Maeda T, Nagata Y, Kitanaka A, Mizuno S, 2016 Aberrant *PD-L1* expression through 3′-UTR disruption in multiple cancers. Nature 534: 402–406. 10.1038/nature18294 27281199

[GR261941SAKC17] Kohno T, Tsuta K, Tsuchihara K, Nakaoku T, Yoh K, Goto K. 2013 *RET* fusion gene: translation to personalized lung cancer therapy. Cancer Sci 104: 1396–1400. 10.1111/cas.12275 23991695 PMC5439108

[GR261941SAKC18] Kryukov GV, Wilson FH, Ruth JR, Paulk J, Tsherniak A, Marlow SE, Vazquez F, Weir BA, Fitzgerald ME, Tanaka M, 2016 *MTAP* deletion confers enhanced dependency on the PRMT5 arginine methyltransferase in cancer cells. Science 351: 1214–1218. 10.1126/science.aad5214 26912360 PMC4997612

[GR261941SAKC19] Li H. 2013 Aligning sequence reads, clone sequences and assembly contigs with BWA-MEM. arXiv:1303.3997 [q-bio.GN].

[GR261941SAKC20] Li H. 2018 Minimap2: pairwise alignment for nucleotide sequences. Bioinformatics 34: 3094–3100. 10.1093/bioinformatics/bty191 29750242 PMC6137996

[GR261941SAKC21] Li H, Handsaker B, Wysoker A, Fennell T, Ruan J, Homer N, Marth G, Abecasis G, Durbin R, 1000 Genome Project Data Processing Subgroup. 2009 The Sequence Alignment/Map format and SAMtools. Bioinformatics 25: 2078–2079. 10.1093/bioinformatics/btp352 19505943 PMC2723002

[GR261941SAKC22] Liu S, Tsai WH, Ding Y, Chen R, Fang Z, Huo Z, Kim S, Ma T, Chang TY, Priedigkeit NM, 2015 Comprehensive evaluation of fusion transcript detection algorithms and a meta-caller to combine top performing methods in paired-end RNA-seq data. Nucleic Acids Res 44: e47 10.1093/nar/gkv1234 26582927 PMC4797269

[GR261941SAKC23] Loman NJ, Quick J, Simpson JT. 2015 A complete bacterial genome assembled *de novo* using only nanopore sequencing data. Nat Methods 12: 733–735. 10.1038/nmeth.3444 26076426

[GR261941SAKC24] Mainardi S, Mulero-Sánchez A, Prahallad A, Germano G, Bosma A, Krimpenfort P, Lieftink C, Steinberg JD, de Wit N, Gonçalves-Ribeiro S, 2018 SHP2 is required for growth of *KRAS*-mutant non-small-cell lung cancer in vivo. Nat Med 24: 961–967. 10.1038/s41591-018-0023-9 29808006

[GR261941SAKC25] Marjon K, Cameron MJ, Quang P, Clasquin MF, Mandley E, Kunii K, McVay M, Choe S, Kernytsky A, Gross S, 2016 *MTAP* deletions in cancer create vulnerability to targeting of the MAT2A/PRMT5/RIOK1 axis. Cell Rep 15: 574–587. 10.1016/j.celrep.2016.03.043 27068473

[GR261941SAKC26] Matsubara D, Kanai Y, Ishikawa S, Ohara S, Yoshimoto T, Sakatani T, Oguni S, Tamura T, Kataoka H, Endo S, 2012 Identification of CCDC6-RET fusion in the human lung adenocarcinoma cell line, LC-2/ad. J Thorac Oncol 7: 1872–1876. 10.1097/JTO.0b013e3182721ed1 23154560

[GR261941SAKC27] Mavrakis KJ, Robert McDonald E, Schlabach MR, Billy E, Hoffman GR, DeWeck A, Ruddy DA, Venkatesan K, Yu J, McAllister G, 2016 Disordered methionine metabolism in MTAP/CDKN2A-deleted cancers leads to dependence on PRMT5. Science 351: 1208–1213. 10.1126/science.aad5944 26912361

[GR261941SAKC28] Mihaylova MM, Shaw RJ. 2012 The AMPK signalling pathway coordinates cell growth, autophagy, and metabolism. Nat Cell Biol 13: 1016–1023. 10.1038/ncb2329 PMC3249400 21892142

[GR261941SAKC29] Mizuguchi T, Suzuki T, Abe C, Umemura A, Tokunaga K, Kawai Y, Nakamura M, Nagasaki M, Kinoshita K, Okamura Y, 2019 A 12-kb structural variation in progressive myoclonic epilepsy was newly identified by long-read whole-genome sequencing. J Hum Genet 64: 359–368. 10.1038/s10038-019-0569-5 30760880

[GR261941SAKC31] Nattestad M, Goodwin S, Ng K, Baslan T, Sedlazeck FJ, Rescheneder P, Garvin T, Fang H, Gurtowski J, Hutton E, 2018 Complex rearrangements and oncogene amplifications revealed by long-read DNA and RNA sequencing of a breast cancer cell line. Genome Res 28: 1126–1135. 10.1101/gr.231100.117 29954844 PMC6071638

[GR261941SAKC30] Nattestad M, Aboukhalil R, Chin C-S, Schatz MC. 2020 Ribbon: intuitive visualization for complex genomic variation. Bioinformatics 10.1093/bioinformatics/btaa680 PMC8058763 32766814

[GR261941SAKC32] Norris AL, Workman RE, Fan Y, Eshleman JR, Timp W. 2016 Nanopore sequencing detects structural variants in cancer. Cancer Biol Ther 17: 246–253. 10.1080/15384047.2016.1139236 26787508 PMC4848001

[GR261941SAKC33] Ohashi A, Ohori M, Iwai K, Nakayama Y, Nambu T, Morishita D, Kawamoto T, Miyamoto M, Hirayama T, Okaniwa M, 2015 Aneuploidy generates proteotoxic stress and DNA damage concurrently with p53-mediated post-mitotic apoptosis in SAC-impaired cells. Nat Commun 6: 7668 10.1038/ncomms8668 26144554 PMC4506520

[GR261941SAKC34] Oikonomopoulos S, Wang YC, Djambazian H, Badescu D, Ragoussis J. 2016 Benchmarking of the Oxford Nanopore MinION sequencing for quantitative and qualitative assessment of cDNA populations. Sci Rep 6: 31602 10.1038/srep31602 27554526 PMC4995519

[GR261941SAKC35] Pleasance ED, Cheetham RK, Stephens PJ, McBride DJ, Humphray SJ, Greenman CD, Varela I, Lin M-L, Ordóñez GR, Bignell GR, 2010 A comprehensive catalogue of somatic mutations from a human cancer genome. Nature 463: 191–196. 10.1038/nature08658 20016485 PMC3145108

[GR261941SAKC36] Poplin R, Ruano-rubio V, Depristo MA, Fennell TJ, Carneiro MO, Van Der Auwera GA, Kling DE, Gauthier D, Levy-moonshine A, Roazen D, 2018 Scaling accurate genetic variant discovery to tens of thousands of samples. bioRxiv 10.1101/201178

[GR261941SAKC37] Rakocevic G, Semenyuk V, Lee W-P, Spencer J, Browning J, Johnson IJ, Arsenijevic V, Nadj J, Ghose K, Suciu MC, 2019 Fast and accurate genomic analyses using genome graphs. Nat Genet 51: 354–362. 10.1038/s41588-018-0316-4 30643257

[GR261941SAKC38] Rizvi H, Sanchez-Vega F, La K, Chatila W, Jonsson P, Halpenny D, Plodkowski A, Long N, Sauter JL, Rekhtman N, 2018 Molecular determinants of response to anti–programmed cell death (PD)-1 and anti–programmed death-ligand 1 (PD-L1) blockade in patients with non–small-cell lung cancer profiled with targeted next-generation sequencing. J Clin Oncol 36: 633–641. 10.1200/JCO.2017.75.3384 29337640 PMC6075848

[GR261941SAKC39] Robinson JT, Thorvaldsdóttir H, Winckler W, Guttman M, Lander ES, Getz G, Mesirov JP. 2011 Integrative Genomics Viewer. Nat Biotechnol 29: 24–26. 10.1038/nbt.1754 21221095 PMC3346182

[GR261941SAKC40] Roth A, Khattra J, Yap D, Wan A, Laks E, Biele J, Ha G, Aparicio S, Bouchard-Côté A, Shah SP. 2014 PyClone: statistical inference of clonal population structure in cancer. Nat Methods 11: 396–398. 10.1038/nmeth.2883 24633410 PMC4864026

[GR261941SAKC41] Ruess DA, Heynen GJ, Ciecielski KJ, Ai J, Berninger A, Kabacaoglu D, Görgülü K, Dantes Z, Wörmann SM, Diakopoulos KN, 2018 Mutant *KRAS*-driven cancers depend on *PTPN11*/SHP2 phosphatase. Nat Med 24: 954–960. 10.1038/s41591-018-0024-8 29808009

[GR261941SAKC42] Sedlazeck FJ, Rescheneder P, Smolka M, Fang H, Nattestad M, von Haeseler A, Schatz MC. 2018 Accurate detection of complex structural variations using single-molecule sequencing. Nat Methods 15: 461–468. 10.1038/s41592-018-0001-7 29713083 PMC5990442

[GR261941SAKC43] Seki M, Katsumata E, Suzuki A, Sereewattanawoot S, Sakamoto Y, Mizushima-Sugano J, Sugano S, Kohno T, Frith MC, Tsuchihara K, 2019 Evaluation and application of RNA-Seq by MinION. DNA Res 26: 55–65. 10.1093/dnares/dsy038 30462165 PMC6379022

[GR261941SAKC44] Seo JS, Ju YS, Lee WC, Shin JY, Lee JK, Bleazard T, Lee J, Jung YJ, Kim JO, Shin JY, 2012 The transcriptional landscape and mutational profile of lung adenocarcinoma. Genome Res 22: 2109–2119. 10.1101/gr.145144.112 22975805 PMC3483540

[GR261941SAKC45] Seo JS, Rhie A, Kim J, Lee S, Sohn MH, Kim CU, Hastie A, Cao H, Yun JY, Kim J, 2016 *De novo* assembly and phasing of a Korean human genome. Nature 538: 243–247. 10.1038/nature20098 27706134

[GR261941SAKC46] Sethi G, Shanmugam MK, Arfuso F, Kumar AP. 2018 Role of RNF20 in cancer development and progression: a comprehensive review. Biosci Rep 38: BSR20171287 10.1042/BSR20171287 29934362 PMC6043722

[GR261941SAKC47] Shi L, Guo Y, Dong C, Huddleston J, Yang H, Han X, Fu A, Li Q, Li N, Gong S, 2016 Long-read sequencing and *de novo* assembly of a Chinese genome. Nat Commun 7: 12065 10.1038/ncomms12065 27356984 PMC4931320

[GR261941SAKC48] Skoulidis F, Goldberg ME, Greenawalt DM, Hellmann MD, Awad MM, Gainor JF, Schrock AB, Hartmaier RJ, Trabucco SE, Gay L, 2018 *STK11/LKB1* mutations and PD-1 inhibitor resistance in *KRAS*-mutant lung adenocarcinoma. Cancer Discov 8: 822–835. 10.1158/2159-8290.CD-18-0099 29773717 PMC6030433

[GR261941SAKC49] Smits AJJ, Kummer JA, De Bruin PC, Bol M, Van Den Tweel JG, Seldenrijk KA, Willems SM, Offerhaus GJA, De Weger RA, Van Diest PJ, 2014 The estimation of tumor cell percentage for molecular testing by pathologists is not accurate. Mod Pathol 27: 168–174. 10.1038/modpathol.2013.134 23887293

[GR261941SAKC50] Suzuki A, Mimaki S, Yamane Y, Kawase A, Matsushima K, Suzuki M, Goto K, Sugano S, Esumi H, Suzuki Y, 2013a Identification and characterization of cancer mutations in Japanese lung adenocarcinoma without sequencing of normal tissue counterparts. PLoS One 8: e73484 10.1371/journal.pone.0073484 24069199 PMC3772023

[GR261941SAKC51] Suzuki M, Makinoshima H, Matsumoto S, Suzuki A, Mimaki S, Matsushima K, Yoh K, Goto K, Suzuki Y, Ishii G, 2013b Identification of a lung adenocarcinoma cell line with CCDC6-RET fusion gene and the effect of RET inhibitors *in vitro* and *in vivo* Cancer Sci 104: 896–903. 10.1111/cas.12175 23578175 PMC7657251

[GR261941SAKC52] Suzuki A, Makinoshima H, Wakaguri H, Esumi H, Sugano S, Kohno T, Tsuchihara K, Suzuki Y. 2014 Aberrant transcriptional regulations in cancers: genome, transcriptome and epigenome analysis of lung adenocarcinoma cell lines. Nucleic Acids Res 42: 13557–13572. 10.1093/nar/gku885 25378332 PMC4267666

[GR261941SAKC53] Suzuki A, Suzuki M, Mizushima-Sugano J, Frith MC, Makałowski W, Kohno T, Sugano S, Tsuchihara K, Suzuki Y. 2017 Sequencing and phasing cancer mutations in lung cancers using a long-read portable sequencer. DNA Res 24: 585–596. 10.1093/dnares/dsx027 29117310 PMC5726485

[GR261941SAKC54] Suzuki A, Kawano S, Mitsuyama T, Suyama M, Kanai Y, Shirahige K, Sasaki H, Tokunaga K, Tsuchihara K, Sugano S, 2018 DBTSS/DBKERO for integrated analysis of transcriptional regulation. Nucleic Acids Res 46: D229–D238. 10.1093/nar/gkx1001 29126224 PMC5753362

[GR261941SAKC55] Thorvaldsdóttir H, Robinson JT, Mesirov JP. 2013 Integrative Genomics Viewer (IGV): high-performance genomics data visualization and exploration. Brief Bioinform 14: 178–192. 10.1093/bib/bbs017 22517427 PMC3603213

[GR261941SAKC56] Treangen TJ, Salzberg SL. 2012 Repetitive DNA and next-generation sequencing: computational challenges and solutions. Nat Rev Genet 13: 36–46. 10.1038/nrg3117 PMC3324860 22124482

[GR261941SAKC57] Tsherniak A, Vazquez F, Montgomery PG, Weir BA, Kryukov G, Cowley GS, Gill S, Harrington WF, Pantel S, Krill-Burger JM, 2017 Defining a cancer dependency map. Cell 170: 564–576.e16. 10.1016/j.cell.2017.06.010 28753430 PMC5667678

[GR261941SAKC58] Wang K, Li M, Hakonarson H. 2010 ANNOVAR: functional annotation of genetic variants from high-throughput sequencing data. Nucleic Acids Res 38: e164 10.1093/nar/gkq603 20601685 PMC2938201

[GR261941SAKC59] Wolman MA, deGroh ED, McBride SM, Jongens TA, Granato M, Epstein JA. 2014 Modulation of cAMP and Ras signaling pathways improves distinct behavioral deficits in a zebrafish model of neurofibromatosis Type 1. Cell Rep 8: 1265–1270. 10.1016/j.celrep.2014.07.054 25176649 PMC5850931

[GR261941SAKC60] Wong GS, Zhou J, Liu JB, Wu Z, Xu X, Li T, Xu D, Schumacher SE, Puschhof J, McFarland J, 2018 Targeting wild-type *KRAS*-amplified gastroesophageal cancer through combined MEK and SHP2 inhibition. Nat Med 24: 968–977. 10.1038/s41591-018-0022-x 29808010 PMC6039276

[GR261941SAKC61] Yoh K, Seto T, Satouchi M, Nishio M, Yamamoto N, Murakami H, Nogami N, Matsumoto S, Kohno T, Tsuta K, 2016 Vandetanib in patients with previously treated *RET*-rearranged advanced non-small-cell lung cancer (LURET): an open-label, multicentre phase 2 trial. Lancet Respir Med 6: 2–10. 10.1016/S2213-2600(16)30322-8 27825616

[GR261941SAKC62] Yokoyama TT, Sakamoto Y, Seki M, Suzuki Y, Kasahara M. 2019 MoMI-G: modular multi-scale integrated genome graph browser. BMC Bioinformatics 20: 548 10.1186/s12859-019-3145-2 31690272 PMC6833150

