# Supplementary material for: Long-read sequencing for non-small-cell lung cancer genomes
Source: Genome Res. 2020 Sep;30(9):1243–57. doi: 10.1101/gr.261941.120 (PMC7545141; doi:10.1101/gr.261941.120)
Supplement: Supplemental Material [file supp_30_9_1243__index.html]

Long-read sequencing for non-small-cell lung cancer genomes — Long-read sequencing for non-small-cell lung cancer genomes — Supplemental Material 

# Long-read sequencing for non-small-cell lung cancer genomes

## Supplemental Material

- Supplemental\_material.docx
- Supplemental\_Files.zip
- Supplemental\_Code.zip
