# Supplementary material for: Long-read sequencing for non-small-cell lung cancer genomes
Source: Genome Res. 2020 Sep;30(9):1243–57. doi: 10.1101/gr.261941.120 (PMC7545141; doi:10.1101/gr.261941.120)
Supplement: Supplemental Material [file supp_gr.261941.120_Supplemental_material.docx]

**Long read sequencing for non-small cell lung cancer genomes**

Yoshitaka Sakamoto, Liu Xu, Masahide Seki, Toshiyuki T. Yokoyama, Masahiro Kasahara, Yukie Kashima, Akihiro Ohashi, Yoko Shimada, Noriko Motoi, Katsuya Tsuchihara, Susumu S. Kobayashi, Takashi Kohno, Yuichi Shiraishi, Ayako Suzuki, Yutaka Suzuki

Supplemental Figures S1 - S13

Supplemental Tables S1- S6

Supplemental Methods

References

### Supplemental Figure S1 General statistics of PromethION data in LC2/ad cells generated from one flow cell

(**A**) Raw read length distribution of LC2/ad PromethION sequencing (one flow cell; 11×). The N50 length and the number of reads >50 kb are shown in the inset. (**B**) General statistics of LC2/ad PromethION sequencing (one flow cell).

### Supplemental Figure S2 General statistics of MinION and PromethION sequencing in four cell lines.

(**A**-**D**) Length distribution of whole-genome sequencing reads (left) and cumulative depth curve of each cell line (right). In the length distribution, the number of reads over 50 kb and N50 length of reads are displayed. (**A**) MinION sequencing of A549 cells. (**B**) PromethION sequencing of RERF-LC-KJ cells. (**C**) PromethION sequencing of RERF-LC-MS cells. (**D**) PromethION sequencing of PC-14 cells. (**E**) Percent sequence identity of the mapped reads in each cell line. KJ: RERF-LC-KJ. MS: RERF-LC-MS. The points indicate the average of sequence identity of each cell line.


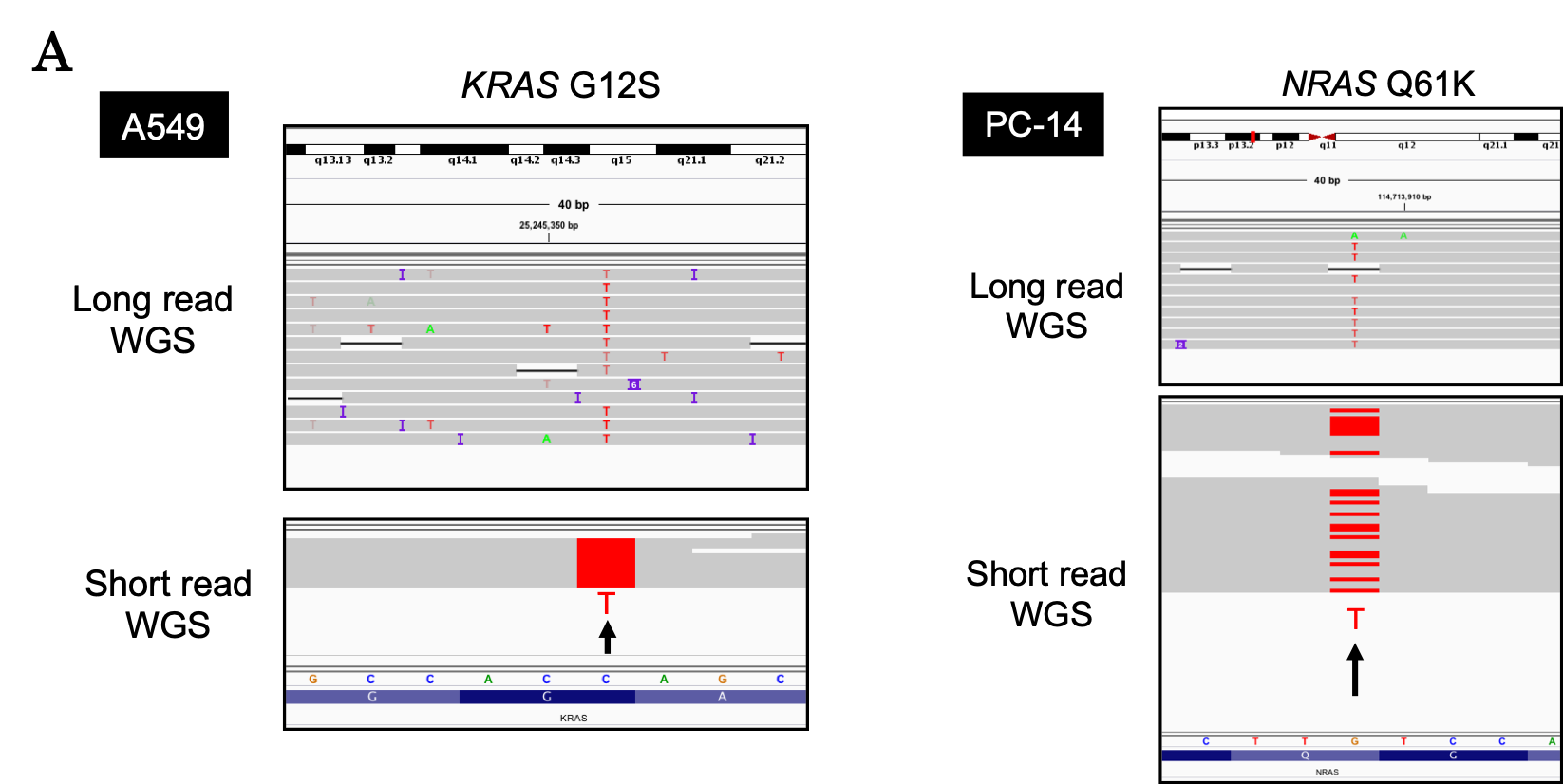

### Supplemental Figure S3 Detection of SNVs in PromethION reads

(**A**) Driver mutations of A549 cells (left) and PC-14 cells (right). IGV images of the point mutations in the driver genes *KRAS* and *NRAS* in MinION/PromethION sequencing (upper) and in Illumina sequencing (lower). *KRAS* point mutation (G12S) is a driver mutation in A549 cells and *NRAS* point mutation (Q61K) is a driver mutation in PC-14 cells. (**B**) Comparison of SNV detection between Illumina and PromethION datasets in LC2/ad. The Venn diagrams of SNV detection with VAF ≥ 0.2 and 0.7 in left and right panels, respectively. Precision and recall rates are presented in the margin. (**C**) The IGV visualization of a known mutation of *TP53* in PromethION reads of LC2/ad. (**D**) SNVs in CDS of tumor suppressor genes. We detected SNVs from both Illumina and PromethION datasets of LC2/ad cells in seven representative tumor suppressor genes (*TP53*, *CDKN1A*, *CDKN2A*, *RB1*, *STK11*, *BRCA1*, and *APC*) and extracted SNVs with the annotation of “exonic.” One of five SNVs was discrepant between the datasets. This SNV was called in initial detection but removed from the Illumina dataset by the filtering step depending on the detection method.

Note: For the general evaluation of SNV detection, we called SNVs in Chromosome 22 from the LC2/ad PromethION dataset using Nanopolish (Loman et al. 2015). Then, we compared the results with those of the Illumina dataset (Suzuki et al. 2014). In total, nearly 90 K and 40 K SNVs were called from the PromethION and Illumina datasets, respectively (precision: 0.33, recall: 0.71 at VAF of ≥ 0.2) as shown in B. The overall detection of SNVs from the PromethION dataset is still considerably noisy because of the limited sequencing accuracy of PromethION. However, when we consider the SNVs with high VAF, which represent homozygous SNVs and/or those existing in the major population of cancer cells, we found that higher detection accuracy can be obtained (precision: 0.79, recall: 0.77 at VAF of ≥ 0.7) as shown in B. This is also consistent with our previous report (Suzuki et al. 2017). For example, a known cancerous mutation of TP53 S241C (100% VAF in Illumina data) was also called from the PromethION dataset as shown in C. See also **Supplemental Figure S3D** for other examples of the SNVs that were called from representative tumor suppressor genes and thus potentially harbor loss-of-function mutations. In total, five exonic SNVs (**D**) were detected in either PromethION or Illumina datasets at VAFs of ≥ 0.7, of which four overlapped between the two approaches. These results collectively indicate that mutation calling at the single-base level is also possible using only the long read sequencer, at least when we detect homozygous SNPs or mutations in LOH regions and the cancer cell contents are as high as in the cultured cells.

### Supplemental Figure S4 IGV images of large deletions detected by MinION and PromethION.

(**A**) A large deletion of *RB1* in RERF-LC-KJ. (**B**) A large deletion of *SMARCA4* in RERF-LC-KJ. (**C**) A large deletion of *STK11* in RERF-LC-MS. These deletions were previously detected using Illumina sequencing.

### Supplemental Figure S5 SV detection of LC2/ad

(**A**) Number of SVs detected in five cell lines by using MinION and PromethION reads. The color code is as shown in the inset. (**B**) Venn diagram of SVs detected from the long reads and short reads of LC2/ad cells. SVs were detected from the PromethION dataset by our computational procedure (see **Methods** and **Supplemental Fig. S8**) and the Illumina dataset by GenomonSV. Briefly, we extracted SVs as follows: 1) 2 kb or larger in size; 2) either of the junctions should be located in genic regions; and 3) SVs should not be detected from the “normal control” panel. (**C**) Venn diagram of the number of SVs detected in LC2/ad cells. SVs were detected from the PromethION and Illumina datasets by Sniffles and by GenomonSV, respectively. We extracted SVs with ≥ 50 bp, excluded insertions, and allowed 100-bp margins for the comparison. (**D**) Number of SVs detected for each of the categories. (**E**) The discrepancies of SV detection at the indicated VAFs. The proportion of the detected SVs from both PromethION and Illumina or the Illumina only are shown in purple and blue, respectively. (**F**) The precision rates of the SV detection in the Illumina dataset. The precision rates at the indicated ranges of VAFs were calculated for all types taken together or separately for each of the SV types, as shown in the left and right panels.

### Supplemental Figure S6 General statistics of PromethION in SK-BR-3 cells

Left: the distribution of read lengths. The inset shows the distribution of read lengths >10 kb. Right: the violin plot of mapping qualities. The dots in the violin plot show the average of sequencing identities.

Note: We generated a total of more than 49 million reads (about 140 Gb, at 46×), with the maximum and N50 lengths of the reads being more than 1 Mb and 20,281 bp, respectively (**Supplemental Table S5**).


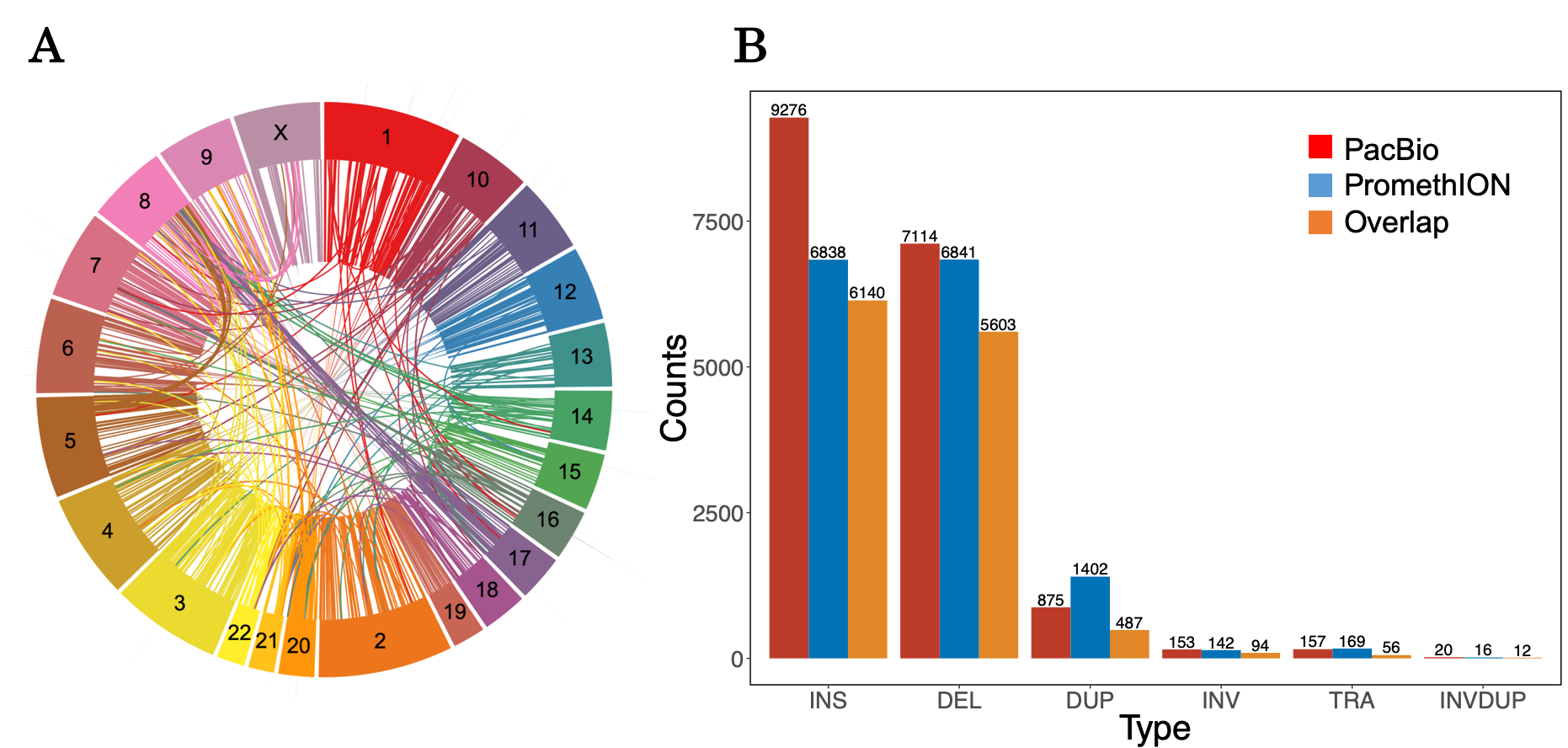

### Supplemental Figure S7 Structural variants detected in SK-BR-3 cells

(**A**) A circos plot of detected SVs using Sniffles >50 bp in SV length. (**B**) The number of six types of SVs detected from each of the datasets. (**C**) The duplication >10 kb in Chromosome 1 only detected from the PromethION dataset. Both the SV junctions were in the gene regions *CHTOP* and *KCNN3*.

Note: To ensure a fair comparison of the detected SVs from PromethION with those from PacBio, we used criteria as similar as possible to those used in the previous study using the PacBio dataset (Nattestad et al. 2018). In particular, the SVs of ≥50 bp in length and translocations/inverted duplications supported by at least 10 reads were selected. Under these criteria, Sniffles detected a total of 15,408 SVs, including 6,838 insertions, 6,841 deletions, 1,402 duplications, 142 inversions, 169 translocations, and 16 inverted duplications from the PromethION dataset.

### Supplemental Figure S8 Pipeline for the detection of complicated local SVs, CLCLs

The detailed procedure is detailed in **Methods**. Briefly, long read sequences in a FASTQ file were mapped to the reference genome hg38 by using minimap2. We clustered reads with split alignment by the IDs of reads. Reads with multiple hits (flag: 256) and <30 MAPQ were filtered out. Junction candidates for SVs were extracted considering the position of the reads and merged allowing 50-bp margins. The junctions supported by ≥5 reads were extracted as SVs in cell lines and clinical samples, respectively. Candidates with less than 2,000 bp between the junctions were discarded. Split alignment means that a read can be split and the subreads can be mapped to different positions in the reference genome.

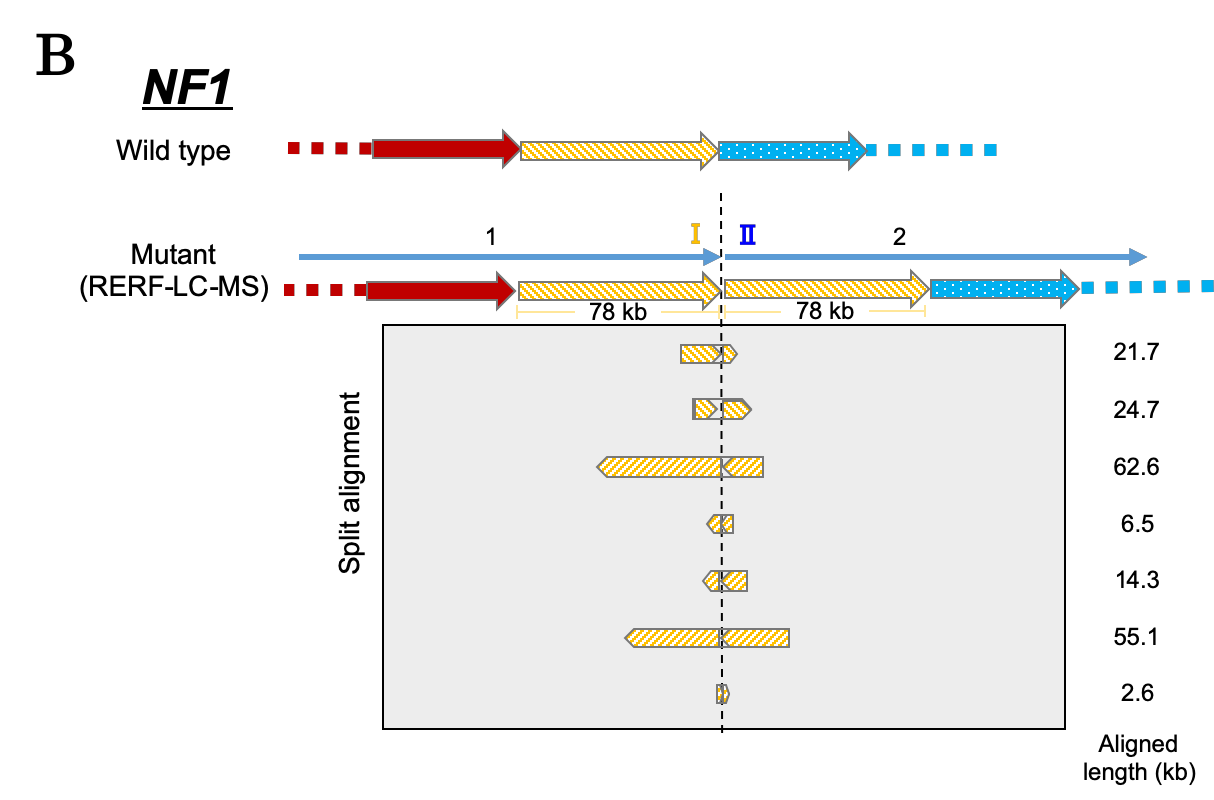

### Supplemental Figure S9 Complicated local SVs represented by PromethION reads

Complicated local SVs in *PTEN* (**A**), *NF1* (**B**), *SMARCA4* (**C**) and *RNF20* (**D**) genes. Split alignments of the reads are shown in the box. The information on the junctions of the SVs is same as shown in **Figure 4**. Forward and reverse strand mapped alignments to the reference genome are represented in red and blue, respectively. (**E-I**) CLCLs represented by RIBBON for *STK11* (**E**), *PTEN* (**F**), *NF1* (**G**), *SMARCA4* (**H**), and *RNF20* (**I**) genes.

### Supplemental Figure S10 Sanger sequencing of the junctions of CLCLs

(**A**) CLCL junctions of the *PTEN* gene in PC-14 were validated by direct Sanger sequencing. The CLCL structure is shown at the top of the panel. PCR and sequencing primers are detailed in the **Methods** section. We detected 2-bp overlaps in both junctions. (**B**) The junction point candidates detected from PromethION reads and those validated by Sanger sequencing.

### Supplemental Figure S11 Histological images of clinical lung cancer specimens

All frozen specimens showed the histology as the final diagnosis: large cell carcinoma (S1), adenocarcinoma with various subtypes [micropapillary in S2, S5, S7, S13, S15, S21, solid in S3, papillary in S6, S8, S10, S18, S19, S20, lepidic in S9, and variant (mixed mucinous and nonmucinous) in S17], squamous cell carcinoma (S11 and S14), pleomorphic carcinoma (S12), and LCNEC (S16). HE staining; S1–S10: bar = 100 µm, S11–S21: bar = 200 µm

### Supplemental Figure S12 Putative CLCLs of cancer-related genes in public datasets

Putative CLCLs of cancer-related genes in 16 LUAD cases (shown as diamond). The driver mutation status of each case is shown at the bottom (blue and red). Gene types indicate driver genes that are significantly functionally altered in the indicated cancer types.

Top -> Download bulk data

-> CLCL (<https://kero.hgc.jp/cgi-bin/download/CLCL>)

- - fastq
  - bam
  - sanger

<https://kero.hgc.jp/examples/CLCL/hg38/>

### Supplemental Figure S13 Data contents in DBKERO

(**A**) The top page and the represented data contents in DBKERO (Suzuki et al. 2018) are shown. In the download page, the FASTQ and BAM files of the whole-genome sequencing data by MinION/PromethION are also downloadable for the cell lines. The sequences were mapped to UCSC hg38 by minimap2 and the resulting BAM files were generated by SAMtools. The chromatogram and the sequence data for the Sanger sequencing is also presented. (**B**) CLCLs cases are visualized in DBKERO. A representative case is shown here. For the CLCLs in cell lines, see genome browser (https://kero.hgc.jp/examples/CLCL/hg38/).

**Supplemental Table S1 Summary of lung cancer cell lines**

| Cell lines | Driver mutation | Ethnicity | Gender |
| --- | --- | --- | --- |
| LC2/ad | *CCDC6-RET* fusion | Japanese | Female |
| A549 | *KRAS* G12S | Non-Asian | Male |
| PC-14 | NRAS Q61K | Japanese | Male |
| RERF-LC-MS | Unknown | Japanese | Male |
| RERF-LC-KJ | Unknown | Japanese | Male |

**Supplemental Table S2 Sequencing statistics of MinION and PromethION**

A549: sequenced by MinION (6 runs); the others: sequenced by PromethION (one run)

**Supplemental Table S3 Candidate novel fusion genes**

**A. LC2/ad**

| Chr1 | Position1 | Chr2 | Position2 | Gene1 | Gene2 | Nanopore reads (MinION, PromethION) | Supporting by  Illumina |
| --- | --- | --- | --- | --- | --- | --- | --- |
| 12 | 3710942 | 20 | 35985933 | *CRACR2A* | *CNBD2* | 0, 8 | No |
| 5 | 107380583 | 8 | 42299774 | *EFNA5* | *IKBKB* | 10, 6 | Yes |
| 11 | 21220687 | 4 | 90671883 | *NELL1* | *CCSER1* | 6, 9 | Yes |
| 11 | 21220704 | 4 | 90671054 | *NELL1* | *CCSER1* | 3, 9 | No |
| 5 | 107378036 | 8 | 42370815 | *EFNA5* | *POLB* | 12, 13 | Yes |
| 12 | 99376310 | 9 | 70623503 | *ANKS1B* | *TRPM3* | 14, 23 | Yes |
| 12 | 99376462 | 9 | 71740824 | *ANKS1B* | *CEMIP2* | 13, 11 | Yes |

**B. RERF-LC-KJ**

| Chr1 | Position1 | Chr2 | Position2 | Gene1 | Gene2 | Nanopore reads | Supporting by  Illumina |
| --- | --- | --- | --- | --- | --- | --- | --- |
| 12 | 52794053 | 9 | 133143828 | *KRT3* | *RALGDS* | 9 | No |
| 12 | 53104293 | 9 | 133346972 | *SOAT2* | *MED22* | 6 | No |
| 15 | 60358715 | 16 | 85612297 | *ANXA2* | *GSE1* | 6 | No |
| 3 | 191,278,568 | 6 | 34,029,376 | *UTS2B* | *GRM4* | 9 | Yes |

**C. PC-14**

| Chr1 | Position1 | Chr2 | Position2 | Gene1 | Gene2 | Nanopore reads | Supporting by  Illumina |
| --- | --- | --- | --- | --- | --- | --- | --- |
| 14 | 90262133 | 9 | 105535792 | *PSMC1* | *FSD1L* | 5 | No |

Nanopore reads: the number of nanopore reads supporting the junctions. In LC2/ad cells, the number of both MinION and PromethION reads are also shown (left, MinION; right, PromethION).

### Supplemental Table S4 Information on SV junctions in LC2/ad

**A. SVs only detected in PromethION**


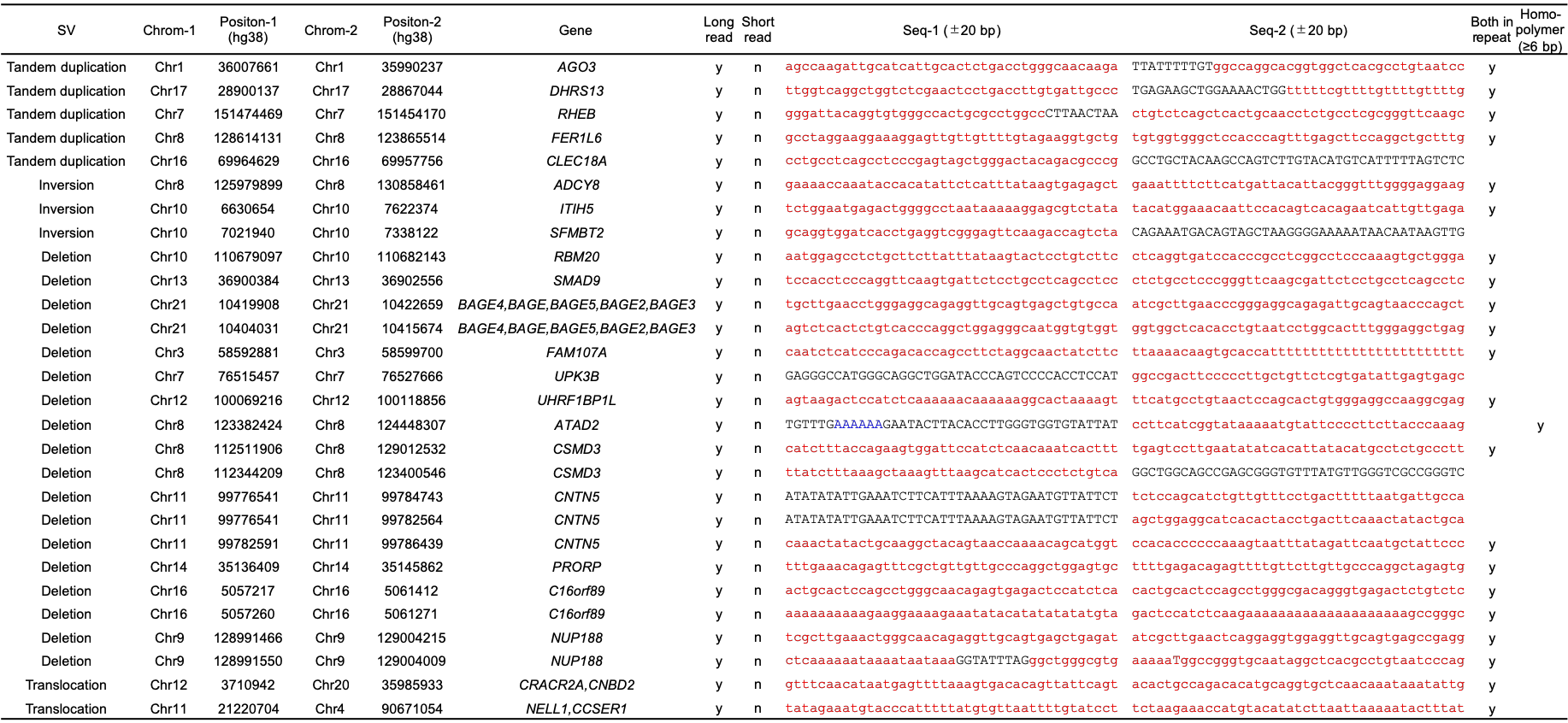


**B. SVs only detected in Illumina**

*One false detection (a deletion in *EDA* gene) was manually discarded from the table because the SV was detected in short read data of normal samples.

Note: We compared SV detection between the short read and long read datasets (>30×) of LC2/ad. We examined the cause of the discrepancy and found that the problems tended to be on the short read side. For example, 75% of the junction points (21/28) that were detected solely from long reads resided in the repeat regions of the human genome (red), where the short reads were not always able to detect the precise structures of the SVs. On the contrary, approximately half of the junction points detected only from short reads were also in the repeat regions (6/14, 48%) and three of the rest resided near a homopolymer (blue), where the short reads might cause the erroneous detection of SVs. A few SVs could not be detected in long reads because of sequencing depths and thresholds of detection parameters.

### Supplemental Table S5 General statistics of nanopore sequencing in SK-BR-3

| Categories | minimap2  (version 2.9) | NGMLR  (version 0.2.7) |
| --- | --- | --- |
| Yields | 139 Gb | - |
| Number of Reads | 49,625,658 | - |
| N50 Length of Reads | 20,281 bp | - |
| Coverage | 46× | - |
| Percentage of Mapped Reads | 79% | 74% |
| Average Length of Mapped Reads | 3,253 bp | 3,269 bp |
| Average Identity of Mapped Reads | 90% | 91% |

**Supplemental Table S6 Information on lung cancer clinical samples**

| Case | Age | Gender | Pathological stage | Histological characters | T/N ratio  (tumor purity) | Necrosis | Inflammation | Driver/oncogenic mutation |
| --- | --- | --- | --- | --- | --- | --- | --- | --- |
| S1 | 76 | Female | IA3 | Large cell carcinoma | Moderate | Present | Mild | Not detected |
| S2 | 57 | Female | IIB | Ad, micropapillary | Moderate | Absent | Moderate | *EGFR* L858R |
| S3 | 54 | Male | IIB | Ad, solid | Moderate | Present | Moderate | *NRAS* Q61L |
| S5 | 38 | Male | IIIA | Ad, micropapillary | Low | Present | Severe | Not detected |
| S6 | 53 | Female | IA3 | Ad, papillary | Moderate | Absent | Moderate | *EGFR* exon 19 deletion |
| S7 | 77 | Female | IA3 | Ad, micropapillary | Moderate | Absent | Moderate | *EGFR* exon 20 insertion |
| S8 | 85 | Female | IB | Ad, papillary | High | Absent | Moderate | *EGFR* exon 19 deletion |
| S9 | 71 | Female | IA1 | Ad, lepidic | Moderate | Absent | Mild | *EGFR* L858R |
| S10 | 65 | Female | IA3 | Ad, papillary | Low | Absent | Severe | *EGFR* L858R |
| S11 | 50 | Male | IIIB | Sq, keratinizing | Moderate | Present | Moderate | Not detected |
| S12 | 85 | Male | IIB | Pleomorphic ca | Low | Absent | Severe | *KRAS* G12C |
| S13 | 59 | Female | IIIA | Ad, micropapillary | Moderate | Absent | Moderate | *EGFR* L858R |
| S14 | 76 | Male | IIIB | Sq, keratinizing | High | Present | Moderate | *PIK3CA* E545K |
| S15 | 78 | Male | IIIA | Ad, micropapillary | Moderate | Absent | Moderate | Not detected |
| S16 | 50 | Male | IIB | LCNEC | High | Present | Severe | Not detected |
| S17 | 68 | Male | IIIA | Ad, variant (mixed mucinous and non-mucinous type) | Moderate | Present | Moderate | Not detected |
| S18 | 61 | Female | IB | Ad, papillary | Moderate | Absent | Moderate to severe | *EGFR* L858R |
| S19 | 55 | Male | IA3 | Ad, papillary | Moderate | Absent | Moderate | Not detected |
| S20 | 70 | Male | IA3 | Ad, papillary | Moderate | Absent | Mild to moderate | *EGFR* exon 19 deletion |
| S21 | 69 | Female | IB | Ad, micropapillary | Moderate | Absent | Moderate | *EGFR* L858R |

*Case S4 was removed from the analysis because of low tumor purity.

### Supplemental Methods

*Library preparation for whole-genome sequencing using MinION*

In summary, 4 µg of HMW gDNA was used for 1D sequencing and DNA repair, end prep, and adapter ligation were conducted. DNA repair was performed using NEBNext FFPE DNA Repair Mix (M6630; NEB). End prep was performed using NEBNext Ultra II End Repair/dA-Tailing Module (E7546L; NEB). Adapter ligation was performed using NEBNext Blunt/TA Ligase Master Mix (M0367L; NEB) and Ligation Sequencing Kit 1D (SQK-LSK108; Oxford Nanopore Technologies). In summary, in 1D^2^ sequencing, 4 or 5 µg of HMW gDNA was used as input, and DNA repair, end prep, first adapter ligation, and second adapter ligation were conducted. DNA repair and end prep were performed following the same protocol as the 1D sequencing. First and second adapter ligations were performed using NEBNext Blunt/TA Ligase Master Mix and Ligation Sequencing Kit 1D^2^ (SQK-LSK308; Oxford Nanopore Technologies). DNA purifications in each step of 1D and 1D^2^ sequencing were performed using Agencourt AMPure XP (A63882; Beckman Coulter). In summary, 15 µl of gDNA was used for rapid sequencing and a Rapid Sequencing Kit (RAD003) was used.

*Library preparation for whole-genome sequencing using PromethION*

To summarize the library preparations for PromethION sequencing, 48 µL of 1.5 or 2 µg of gDNA plus nuclease-free water (NFW), 3.5 µL of NEBNext FFPE DNA Repair Buffer, 2 µL of NEBNext FFPE DNA Repair Mix (M6630; NEB), 3.5 µL of NEBNext Ultra II End Prep Reaction Buffer, and 3 µL of NEBNext Ultra II End Prep Enzyme Mix (E7545L; NEB) were mixed gently in a 1.5 mL Eppendorf tube. After spinning down, the sample was incubated at 20°C for 5 min and 65°C for 5 min. Then, 60 µL of AMPure XP beads (A63882; Beckman Coulter) were added to the tube and the tube was mixed by flicking. The sample was incubated for 5 min at room temperature (R.T.) using a rotator mixer. After spinning down, the tube was set on a magnetic stand and the supernatant was pipetted off. The beads were washed twice with 200 µL of 70% ethanol. The tube was spun down and placed back on the magnet. Any residual ethanol was pipetted off and the tube was dried for 30 s. The tube was removed from the magnetic stand, the pellet was resuspended in 61 µL of NFW, and the tube was incubated for 2 min. The tube was set on a magnet, 60 µL of the sample was used in the next step, and 1 µL was used for a quality check by Qubit. Then, 60 µL of end-prepped DNA, 25 µL of Ligation Buffer (LNB), 10 µL of NEBNext Quick T4 DNA Ligase (NEB, E6056S), and 5 µL of Adapter Mix (AMX) were mixed in a 1.5 mL Eppendorf tube. After spinning down, the sample was incubated at R.T. for 10 min. Then, 40 µL of AMPure XP beads were added to the tube and mixed by flicking. The sample was incubated for 5 min at R.T. using a rotator mixer. After spinning down, the tube was set on the magnetic stand. The supernatant was pipetted off and the beads were washed twice with 250 µL of Fragment Buffer (LFB). The sample was spun down and the tube was placed back on the magnet. Any residual supernatant was pipetted off and the tube was dried for 30 s. The tube was then removed from the magnet and the pellet was resuspended in 25 µL of Elution Buffer (EB). The sample was incubated at R.T. for 10 min. Next, 24 µL of the sample was used in the next step and 1 µL was used for a quality check by Qubit. A total of 46 µL of Flush Tether (FLT) was added directly to the tube of PromethION Flush Buffer (PFB) and the solution was mixed by pipetting (Priming Mix). Then, 800 µL of Priming Mix was loaded onto the PromethION flow cell. Next, 75 µL of SQB, 51 µL of LB, and 24 µL of the DNA library were mixed in a 1.5 mL Eppendorf tube in 5 min (Loading Library). Then, 200 µL of Priming Mix was loaded onto the flow cell and 150 µL of Loading Library was loaded onto the flow cell. We started the PromethION run. The LNB, AMX, LFB, EB, FLT, PFB, SQB, and LB are in Ligation Sequencing Kit 1D (SQK-LSK109; Oxford Nanopore Technologies). For the LC2/ad cells, we performed five runs, with each throughput being 10, 9, 33, 29, and 19 Gb.

*Analysis of SVs using short read sequencing data*

For comparison of SV junctions between long read data (PromethION; our computational procedure) and short read data (Illumina; GenomonSV) from LC2/ad (**Supplemental Fig. S5B**), we allowed 100-bp margins after performing conversion of the reference genome version (https://genome.ucsc.edu/cgi-bin/hgLiftOver). We also extracted and compared SVs with the following conditions: 1) 2 kb or larger SVs, 2) either junction in genic regions, and 3) not observed in control panels that were constructed from ICGC/TCGA data.

For comparison of SV junctions between long read data (PromethION; Sniffles) and short read data (Illumina; GenomonSV) from LC2/ad (**Supplemental Fig. S5C**), we extracted only 50-bp or larger SVs and allowed 100-bp margins for the comparison after performing conversion of the reference genome version.

For **Supplemental Figure S12**, we also performed GenomonSV using 514 whole-exome datasets from TCGA-LUAD and 97 whole-exome datasets from Japanese patients with lung adenocarcinoma (JGAS00000000001) to detect SVs in a manner similar to that mentioned above. GenomonSV filt was performed with the options “--min_junc_num 1” and “--non_matched_control_junction” with control panels that were constructed from ICGC/TCGA data. We also set the option “--matched_control_bam.” After GenomonSV filt, we eliminated SV candidates with tumor VAF < 0.05 and a distance of less than 2 kb between the junctions. We analyzed at least one SV junction within genic regions. Genes with putative CLCLs are defined as those affected by SV junctions with a tandem duplication structure. For CLCLs in cancer-related genes, we checked 299 cancer-related genes that were previously reported as cancer driver genes. The cancer driver genes were classified into gene types as follows: genes significant in lung adenocarcinoma and pan-cancer types as “LUAD and multiple cancer types,” genes significant in multiple and pan-cancer types as “Multiple cancer types,” and genes significant in other cancer types besides lung adenocarcinoma as “Unique to other cancer types” (Bailey et al. 2018). For known driver mutation status, 13 genes (*EGFR*, *KRAS*, *BRAF*, *HRAS*, *NRAS*, *RET*, *MAP2K1*, *ALK*, *ROS1*, *ERBB2*, *MET*, *NF1*, and *RIT1*) were examined in the TCGA cases by cBioPortal (lung adenocarcinoma; TCGA; PanCancer Atlas) (Gao et al. 2013; Cerami et al. 2012; Hoadley et al. 2018).

*Sanger sequencing for validation of the CLCL junctions*

The genomic DNA fragments around the junctions were amplified by PCR using the sets of primers that were designed as shown below: *STK11*-junction I/III: 5′-CCTGAGGAACAGTACACGCC-3′; 5′-CTGCATCCAGGAGATCTGGTC-3′, *STK11*-junction II/IV: 5′-GCTCTGGATTGGCTTGTCCTGGA-3′; 5′-CAAGCAGGAGTGTGCGGTCAATAT-3′, *PTEN*-junction II/III: 5′-GAAGGACTTGGGATAGTCCA-3′; 5′-TGGAGATCTGTACATACAGT-3′, *PTEN*-junction I/IV: 5′-AACATATTCCAAAGACAGAATAGA-3′; 5′-CAGGACTAGATGAGAACCAACCC-3′. The specificity of primers was confirmed with Primer-BLAST in NCBI. The amplification reaction was performed using the KAPA HiFi HotStart ReadyMix (KAPA Biosystems), in accordance with the following program: 98°C for 30 s; 30 cycles of 98°C for 30 s, 62–65°C (annealing temperatures depend on the primer sets, 65°C for *STK11*-junction I/III and *STK11*-junction II/IV, 63°C for *PTEN*-junction II/III, and 62°C for *PTEN*-junction I/IV) for 30 s, 72°C for 30 s (60 s for *STK11*-junction I/III); and then 72°C for 10 min. All the PCR products were confirmed as single peaks using Bioanalyzer (Agilent Technologies) and then purified with AMPure XP beads (Beckman Coulter) to remove primer fragments. The purified PCR fragments were outsourced to a company (FASMAC Co., Ltd.) for Sanger sequencing using Applied Biosystems 3730xl DNA Analyzer.

For the *STK11*-junction I/III, TA cloning was conducted because the sequences around the junction included poly-T sites and could not be elucidated with forward by PCR amplicons of direct Sanger sequencing. The PCR products were cloned using the Mighty TA-cloning Kit (Takara). We attempted to sequence cloned fragments and used the sequencing primer F: 5’-AAAACGCTGGGATGACAGGA-3’ for the chromatogram of *STK11*-junction I/III in **Figure 3C**.

*Western blotting*

For the Western blotting, we performed protein extraction and quantification. We used a Pierce BCA Protein Assay kit (23225; Thermo Fisher Scientific) and prepared 1 mg/ml proteins. In summary, we performed electrophoresis of proteins, membrane transfer, blocking, reaction of the first antibody, reaction of the second antibody, and visualization of bands. We conducted electrophoresis using a tank and used 10 or 15 µl of proteins as input. For blocking, we used 4% BSA blocking buffer. In the reaction of the first antibody, we used LKB1 (27D10) rabbit mAb (3050S; CST) for STK11, phospho-AMPK-alpha(Thr172) antibody (2535S; CST) for phospho-PRKAA1/2, AMPK-alpha antibody rabbit mAb (2603S; CST) for PRKAA1/2, anti-neurofibromin (NF1) (rabbit polyclonal IgG) (07-730; Upstate) for NF1, phospho-p44/42 MAPK (Erk1/2) (Thr202/Thr204) (E10) mouse mAb (9106S; CST) for phospho-MAPK1/3, p44/42 MAPK (Erk1/2) antibody (9102S; CST) for MAPK1/3, Brg1 (D1Q7F) rabbit mAb (49360; CST) for SMARCA4, PTEN (138G6) rabbit mAb (CST; 9559S) for *PTEN* protein, phospho-Akt (Ser473) (D9E) XP rabbit mAb (4060S; CST) for phospho-AKT1/2/3, and Akt (pan) (11E7) rabbit mAb (4685S; CST) for AKT1/2/3. As a control, we used GAPDH and GAPDH (14C10) rabbit mAb (2118S; CST) as an antibody. In the reaction of the second antibody, we used an antibody corresponding to the animal that generated the first antibody. We used anti-rabbit IgG, HRP-linked antibody (7074S, CST) for rabbit and anti-mouse IgG, HRP-linked antibody (7076S, CST) for mouse. For the visualization of bands, we used ImageQuant LAS 4000 Mini (GE Healthcare). The normalized ratio indicated the fraction of the density of phosphorylated protein relative to the control protein and the density of total protein relative to control protein, and we set the normalized ratio of the wild type as 1 (**Fig. 4E**). The density of the bands was calculated using ImageJ software (Schneider et al. 2012).

### References

Bailey MH, Tokheim C, Porta-Pardo E, Sengupta S, Bertrand D, Weerasinghe A, Colaprico A, Wendl MC, Kim J, Reardon B, et al. 2018. Comprehensive Characterization of Cancer Driver Genes and Mutations. *Cell* **173**: 371-385.e18.

Cerami E, Gao J, Dogrusoz U, Gross BE, Sumer SO, Aksoy BA, Jacobsen A, Byrne CJ, Heuer ML, Larsson E, et al. 2012. The cBio cancer genomics portal: an open platform for exploring multidimensional cancer genomics data. *Cancer Discov* **2**: 401–4.

Gao J, Aksoy BA, Dogrusoz U, Dresdner G, Gross B, Sumer SO, Sun Y, Jacobsen A, Sinha R, Larsson E, et al. 2013. Integrative analysis of complex cancer genomics and clinical profiles using the cBioPortal. *Sci Signal* **6**: pl1.

Hoadley KA, Yau C, Hinoue T, Wolf DM, Lazar AJ, Drill E, Shen R, Taylor AM, Cherniack AD, Thorsson V, et al. 2018. Cell-of-Origin Patterns Dominate the Molecular Classification of 10,000 Tumors from 33 Types of Cancer. *Cell* **173**: 291-304.e6.

Loman NJ, Quick J, Simpson JT. 2015. A complete bacterial genome assembled de novo using only nanopore sequencing data. *Nat Methods* **12**: 733–735.

Nattestad M, Goodwin S, Ng K, Baslan T, Sedlazeck FJ, Rescheneder P, Garvin T, Fang H, Gurtowski J, Hutton E, et al. 2018. Complex rearrangements and oncogene amplifications revealed by long-read DNA and RNA sequencing of a breast cancer cell line. *Genome Res* **28**: 1126–1135.

Schneider CA, Rasband WS, Eliceiri KW. 2012. NIH Image to ImageJ: 25 years of Image Analysis HHS Public Access. *Nat Methods* **9**: 671–675.

Suzuki A, Kawano S, Mitsuyama T, Suyama M, Kanai Y, Shirahige K, Sasaki H, Tokunaga K, Tsuchihara K, Sugano S, et al. 2018. DBTSS/DBKERO for integrated analysis of transcriptional regulation. *Nucleic Acids Res* **46**: D229–D238.

Suzuki A, Makinoshima H, Wakaguri H, Esumi H, Sugano S, Kohno T, Tsuchihara K, Suzuki Y. 2014. Aberrant transcriptional regulations in cancers: Genome, transcriptome and epigenome analysis of lung adenocarcinoma cell lines. *Nucleic Acids Res* **42**: 13557–13572.

Suzuki A, Suzuki M, Mizushima-Sugano J, Frith MC, Makałowski W, Kohno T, Sugano S, Tsuchihara K, Suzuki Y. 2017. Sequencing and phasing cancer mutations in lung cancers using a long-read portable sequencer. *DNA Res* **24**: 585–596.
